# ASO Therapy Targeting STAU2 to Inhibit Pancreatic Ductal Adenocarcinoma Progression and Metastasis by Regulating the PALLD‐Mediated EMT Signaling Pathway

**DOI:** 10.1002/advs.202506718

**Published:** 2025-06-20

**Authors:** Jiayu Ding, Hao Shen, Jiaying Ji, Jiaxing Li, Zhongrui Shi, Xuejiao Wang, Bangbang Li, Yi Hou, Wenjian Min, Chengliang Sun, Kai Yuan, Yasheng Zhu, Liping Wang, Shun‐Qing Liang, Wenbin Kuang, Xiao Wang, Peng Yang

**Affiliations:** ^1^ State Key Laboratory of Natural Medicines China Pharmaceutical University Nanjing 210009 China; ^2^ Department of Medicinal Chemistry School of Pharmacy China Pharmaceutical University Nanjing 211198 China; ^3^ Department of Medicine University of Minnesota Twin Cities Minneapolis MN 55455 USA; ^4^ Institute of Innovative Drug Discovery and Development China Pharmaceutical University Nanjing 211198 China

**Keywords:** anti‐sense oligonucleotide, EMT, PALLD, pancreatic ductal adenocarcinoma, STAU2

## Abstract

Pancreatic ductal adenocarcinoma (PDAC) is a highly aggressive malignancy marked by high morbidity, recurrence, and metastasis, with limited treatment options and poor prognosis. The challenge of early diagnosis and the inefficacy of current therapeutic strategies underscore the urgent need for novel biomarkers and therapeutic targets. RNA‐binding proteins (RBPs) are emerging as critical regulators of post‐transcriptional processes and are implicated in cancer progression. Here, the study identifies Staufen Double‐Stranded RNA Binding Protein 2 (STAU2) as an oncogenic RBP with high expression in PDAC, which is significantly associated with metastasis. It is demonstrated that STAU2 directly binds and regulates cytoskeletal associated protein Palladin (PALLD) and mediates IQ motif containing GTPase‐activating protein 1 (IQGAP1), thereby promoting metastasis via the epithelial‐mesenchymal transition (EMT) pathway. Moreover, a 2′‐methoxyethoxy (2′‐MOE)‐modified antisense oligonucleotide (ASO) targeting STAU2 is developed, which effectively inhibited downstream targets in vitro and in vivo. STAU2‐ASO treatment significantly suppressed PDAC progression and metastasis, with a demonstrated safety profile in vivo.

## Graphical abstract

This study identifies STAU2 as an oncogenic RNA‐binding protein (RBP) with elevated expression in pancreatic ductal adenocarcinoma (PDAC), where its high levels are significantly correlated with metastatic progression. It elucidates that STAU2 directly binds and regulates PALLD, while mediating the functional role of IQGAP1, thereby driving metastasis through the EMT pathway. Moreover, STAU2‐ASO treatment inhibits downstream targets in vitro and in vivo, suppresses PDAC progression and metastasis, and demonstrates in vivo safety.

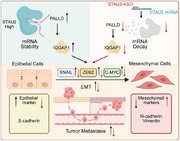

## 1 Introduction

PDAC is among the most aggressive malignancies of the digestive system with a steadily increasing annual incidence.^[^ ^1^, ^2^, ^3^ ^]^ The majority of PDAC cases are diagnosed at an advanced stage, precluding the possibility of surgical intervention.^[^ ^4^ ^]^ Furthermore, recurrences and metastases are frequent even with surgical resection.^[^ ^5^, ^6^ ^]^ These challenges underscore the urgent need for an in‐depth understanding of PDAC pathogenesis and the identification of novel prognostic biomarkers and therapeutic targets to improve patient outcomes.

Recent studies have highlighted the pivotal role of RBPs in reshaping the transcriptomic and proteomic landscapes of tumor cells, thereby influencing the proliferative and metastatic capabilities of multiple tumor types.^[^ ^7^, ^8^, ^9^, ^10^ ^]^ RBPs interact selectively with various RNA species, including rRNA,^[^ ^11^, ^12^, ^13^, ^14^ ^]^ ncRNA,^[^ ^15^, ^16^ ^]^ miRNA^[^ ^17^, ^18^, ^19^ ^]^ and mRNA,^[^ ^20^, ^21^ ^]^ facilitating the formation of ribonucleoprotein complexes^[^ ^22^, ^23^, ^24^ ^]^ and modulating RNA subcellular localization^[^ ^25^, ^26^ ^]^ and translation.^[^ ^24^, ^27^ ^]^ Consequently, unraveling the biological functions of RBPs is crucial for advancing our understanding of RNA‐mediated disease pathogenesis.

STAU2 is a key RBP that is involved in the regulation of mRNA translation and stability.^[^ ^28^, ^29^ ^]^ STAU2 comprises multiple functional domains, including RNA‐binding domain (RBD), tubulin‐binding domain, and Staufen‐swapping motif.^[^ ^30^, ^31^, ^32^ ^]^ RBDs enable STAU2 to recognize specific RNA structures and to interact with downstream targets,^[^ ^32^, ^33^ ^]^ modulating mRNA degradation^[^ ^29^, ^30^ ^]^ and translation.^[^ ^28^, ^29^, ^34^ ^]^ Our previous study,^[^ ^35^ ^]^ we identified STAU2 as a high‐risk RBP associated with elevated expression and poor prognosis in PDAC. However, the precise mechanisms by which STAU2 regulates PDAC progression remain unknown.

In the present study, bioinformatics analysis of tumor genome databases and immunofluorescence staining of clinical tissues demonstrated that STAU2 overexpression was closely associated with PDAC metastasis and correlated with poor overall survival (OS). Notably, STAU2 knockdown resulted in significant tumor growth and metastasis suppression, mediated via down‐regulation of the EMT signaling pathway both in vitro and in vivo. RNA sequencing and immunoprecipitation results identified PALLD as a novel downstream STAU2 target involved in STAU2‐mediated metastasis in PDAC by regulating IQGAP1. A 2′‐ MOE modified antisense oligonucleotide STAU2‐ASO was designed to further explore the therapeutic potential of STAU2. It effectively and specifically suppressed STAU2 expression in vitro, demonstrating efficacy in inhibiting PDAC growth and metastasis in vivo while maintaining a favorable safety profile. Our study findings highlight the significant potential of STAU2 blockade in tumor progression and metastasis, offering a promising perspective and target for the development of innovative anticancer therapies.

## 2 Results

### 2.1 STAU2 Up‐Regulation in PDAC is Associated with Tumor Progression, Metastasis, and Poor Prognosis

**Figure 1:**
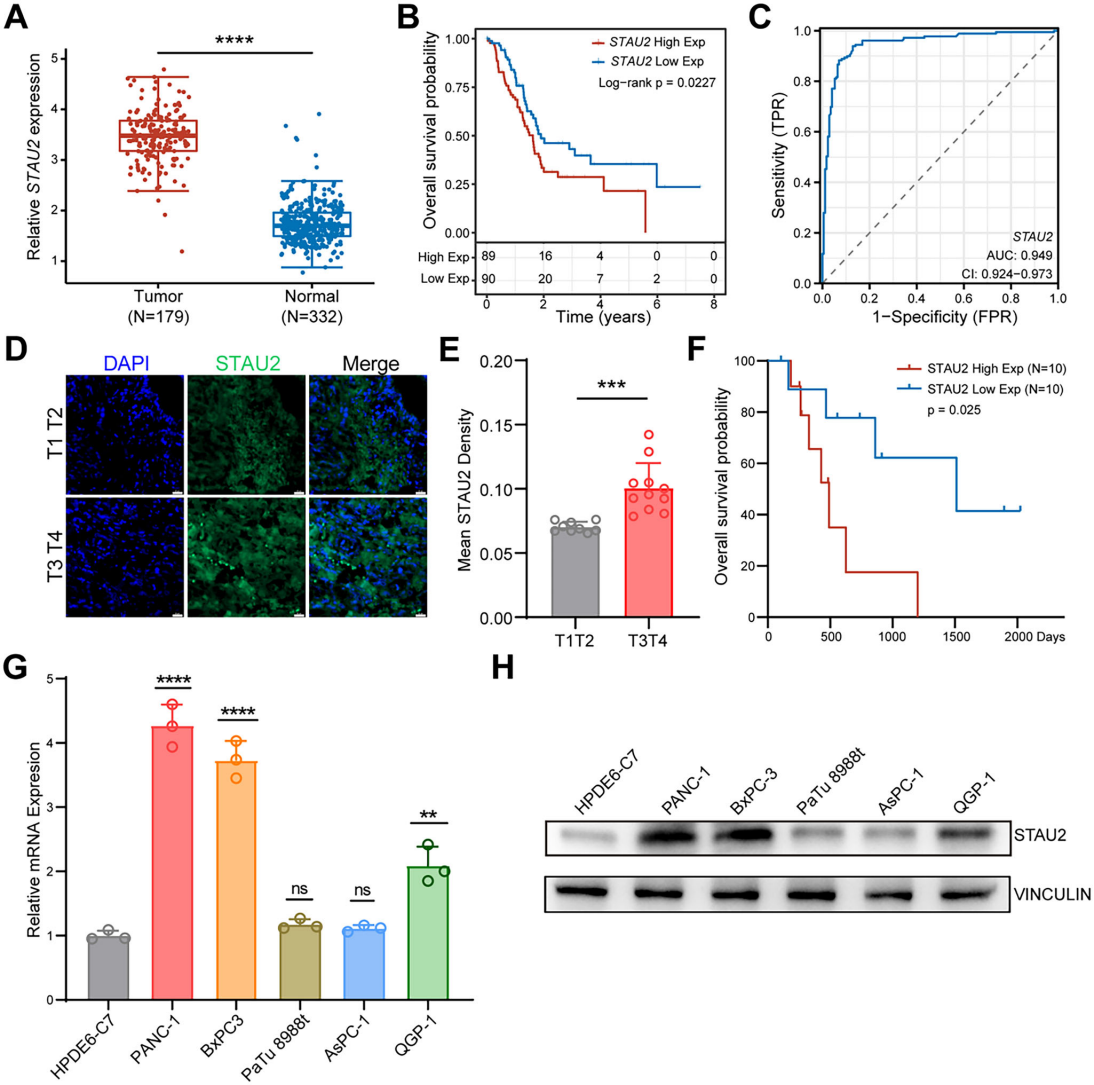
STAU2 is highly expressed in metastatic PDAC and associated with poor survival. A) Comparison of STAU2 expression between PDAC tumor samples (n = 179, from TCGA) and normal samples (n = 332, from TCGA & GTEx). Statistical analysis was performed using wilcoxon test. B) Overall survival Kaplan‐Meier curve of STAU2 high expression (red line, n = 89) and STAU2 low expression (blue line, n = 90) PDAC patient samples (data from TCGA PDAC patients). Statistical analysis was performed using log‐rank test, *p* = 0.0227. C) Receiver operating characteristic (ROC) curve evaluating the diagnostic potential of STAU2 expression in PDAC, AUC = 0.949. D) Immunofluorescence staining of tissue microarrays from PDAC patients using STAU2 antibody. Scale bar, 20 µm. E) Differential ratio of STAU2 mean density in tissue microarray of clinical samples of PDAC at different stages (T_1_T_2_ n = 11, T_3_T_4_ n = 9). Data represent the mean ± SD. Statistical analysis was performed using two‐tailed unpaired student’ s *t*‐test. F) Overall survival Kaplan‐Meier curve of STAU2 high expression (red line, n = 10) and low (blue line, n = 10) STAU2 expression PDAC patient samples. Statistical analysis was performed using log‐rank test, *p* = 0.025. G) RT‐qPCR analysis of STAU2 mRNA expression in PANC‐1, BxPC3, PaTu8988t, AsPC‐1, QGP‐1 cells and human normal pancreas cell HPDE6‐C7. Data represent the mean ± SD, n = 3. Statistical analysis was performed using two‐tailed unpaired student’ s *t*‐test. H) Western blot analysis of STAU2 protein levels in PANC‐1, BxPC3, PaTu8988t, AsPC‐1, QGP‐1 cells and human normal pancreas cell HPDE6‐C7. ^*^, *p* < 0.05; ^**^, *p* < 0.01; ^***^, *p* < 0.001; ^****^, *p* < 0.0001.

It was previously suggested that STAU2 is a potential risk factor for PDAC.^[^ ^35^ ^]^ To further explore the clinical significance of STAU2 in PDAC, STAU2 expression in tumors was compared to that in normal samples using TCGA database. The results revealed a statistically significant STAU2 up‐regulation in tumor samples (**Figure** **1**A). The Kaplan‐Meier survival analysis demonstrated that patients with high levels of STAU2 expression exhibited significantly poorer OS compared to patients with lower expression levels (Figure 1B). Subsequently, the diagnostic potential of STAU2 in PDAC progression was evaluated by analyzing the receiver operating characteristic curve. The area under the curve (AUC) value of 0.949 (Figure 1C) indicated the high diagnostic accuracy of STAU2. Further analysis of Stau2 expression between primary and metastatic PDAC using GSE162791 revealed higher Stau2 levels in metastatic PDAC (Figure S1A, Supporting Information). Furthermore, single‐cell sequencing data from GSE154778 were divided into primary and metastatic PDAC groups in order to analyze STAU2 expression. The results showed that STAU2 was highly expressed in total cell samples of metastatic PDAC (Figure S1C, Supporting Information). Additionally, STAU2 level examination in PDAC microarrays containing 20 tissue samples showed that high STAU2 expression was positively associated with PDAC progression (T_3–4_ vs T_1–2_) and poor survival (Figure 1D–F and Figure S1B, Supporting Information).Real‐Time Quantitative PCR (RT‐qPCR) and western blot analyses were performed to validate STAU2 expression levels and assess their expression in normal pancreatic HPDE6‐C7 and PDAC cell lines, including PANC‐1, BxPC3, PaTu 8988t, ASPC‐1, and QGP‐1 (Figure 1G,H). The results verified the high expression of STAU2 in PDAC cells, with particularly pronounced increase observed in PANC‐1 and BxPC3 cells. Collectively, these findings suggested that STAU2 is a prognostic risk factor and a viable therapeutic target for PDAC.

### 2.2 STAU2 is an Important Factor that Promotes PDAC Cell Proliferation and Metastasis In Vitro and In Vivo

**Figure 2:**
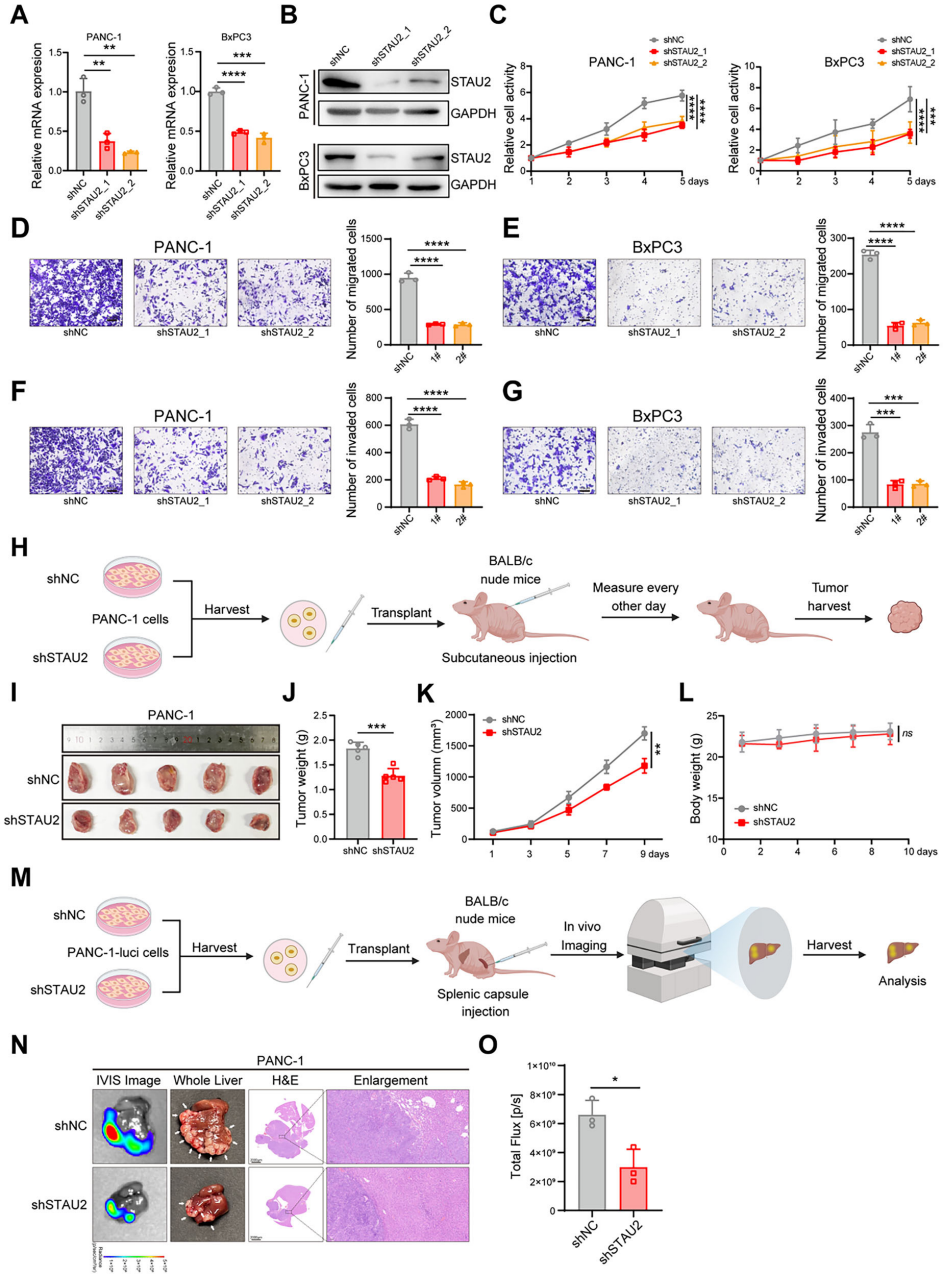
STAU2 deficiency inhibits PDAC proliferation and metastasis in vitro and in vivo. A) RT‐qPCR analysis of STAU2 mRNA expression in PANC‐1 and BxPC3 cells transfected with shNC and two independent shSTAU2 constructs. B) Western blot analysis of STAU2 protein level in PANC‐1 and BxPC3 cells transfected with shNC and two independent shSTAU2 constructs. C) Cell viability of PANC‐1 cells and BxPC3 cells with shNC and two independent shSTAU2 constructs during a 5‐day course. D,E) Migration ability of PANC‐1 (D) and BxPC3 (E) cells with shNC and two independent shSTAU2 constructs. Scale bar, 100 µm. F,G) Invasion ability of PANC‐1 (F) and BxPC3 (G) cells with shNC and two independent shSTAU2 constructs. Representative images of migrated and invaded cells were shown. Scale bar, 100 µm. H–K) BALB/c nude mice subcutaneously transplanted with shSTAU2 cells and shNC PANC‐1 cells. Tumor volumes were measured every 2 days (K), and after mice were euthanized, tumors were excised, photographed (I) and weight (J). L) No significant difference in the body weight of mice between shNC group and shSTAU2 group during the experimental period. Data represent the mean ± SD, n = 5 mice in each group. M,O) Representative bioluminescent images (N, column of IVIS image), photographs (N, column of whole liver, arrows point to the tumor nodules) and HE staining with enlargement (N, columns of HE and enlargement) are shown, respectively. Scale bar, 2000 µm (H&E); 100 µm (enlargement). And quantified after tumor formation in model of PDAC with liver metastasis (O). Data represent the mean ± SD, n = 3. Statistical analysis was performed using two‐tailed unpaired student's *t*‐test. ^*^, *p* < 0.05; ^**^, *p* < 0.01; ^***^, *p* < 0.001; ^****^, *p* < 0.0001.

To investigate the role of STAU2 in PDAC progression, STAU2 knockdown cells were established and confirmed at RNA and protein levels using RT‐qPCR and western blotting (**Figure** **2**A,B). Cell proliferation assays revealed that STAU2 knockdown significantly inhibited PDAC cell proliferation (Figure 2C). Furthermore, the effect of STAU2 on cell invasion and migration was examined using transwell assays. The number of migrating (Figure 2D,E) and invading (Figure 2F,G) tumor cells was significantly reduced after STAU2 knockdown. To assess the functional role of STAU2 in tumor proliferation and metastasis in vivo, BABL/c nude mice were utilized to establish a xenograft model using PANC‐1 cells with STAU2 knockdown (shSTAU2) or non‐targeting control cells (shNC) via subcutaneous injection. Tumor volumes in mice were measured and recorded every two days during the experimental period (Figure 2H–L). Tumor growth in the STAU2 knockdown group was significantly reduced compared to that in the control group, demonstrating an inhibition rate of 30.66% (Figure 2I–K). Additionally, no statistically significant disparity in body weight was observed between the two groups (Figure 2L). Similar results were noted in the BxPC3 xenograft model (Figure S1D–G, Supporting Information). STAU2 silencing significantly inhibited the growth of pancreatic cancer cells, with an inhibition rate of 53.26% in the BxPC3 xenograft model. These results suggested that STAU2 knockdown significantly inhibited tumor growth in vivo. To elucidate the impact of STAU2 in PDAC metastasis in vivo, a liver metastasis model was subsequently established via splenic capsule injection of PANC‐1‐luci cells (Figure 2M). Bioluminescence imaging of the liver showed that the number of tumor nodules was significantly reduced in the shSTAU2 PANC‐1 group compared to that in the shNC group, and the signal intensity was significantly decreased in the shSTAU2 PANC‐1 group (Figure 2N,O). Similar results were observed in the BxPC3 xenograft model (Figure S1H,I, Supporting Information).

**Figure 3:**
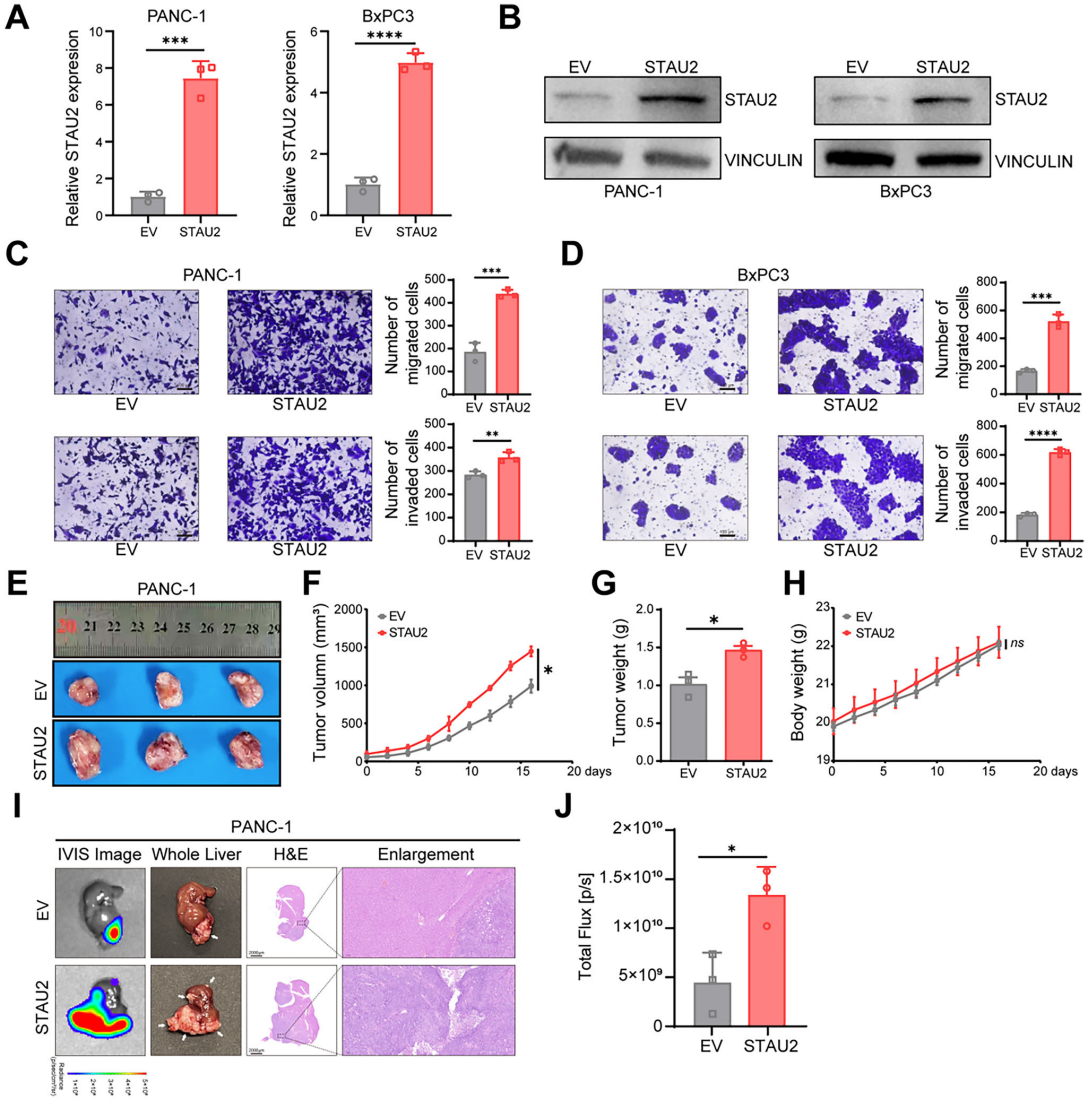
STAU2 overexpression promotes PDAC proliferation and metastasis in vitro and in vivo. RT‐qPCR analysis of STAU2 mRNA expression in PANC‐1 and BxPC3 cells transfected with EV (empty vector)and STAU2 (vector with STAU2 overexpression) constructs. B) Western blot analysis of STAU2 protein level in PANC‐1 and BxPC3 cells that were overexpressed with EV and STAU2. C,D) Migration and invasion ability of PANC‐1 cells (C) and BxPC3 cells (D) that were overexpressed with EV and STAU2. Scale bar, 100 µm. E–H) BALB/c nude mice subcutaneously transplanted with PANC‐1 NC and PANC‐1 STAU2 cells. Tumor volumes were measured every 2 days (F), and after mice were euthanized, tumors were excised, photographed (E) and weight (G). (H) No significant difference in the body weight of mice between NC group and STAU2 group during the experimental period. I) Representative bioluminescent images (column of IVIS image), photographs (column of whole liver, arrows point to the tumor nodules) and HE staining with enlargement (columns of HE and enlargement) are shown, respectively. Scale bar, 2000 µm (H&E); 100 µm (enlargement). J) Quantified after tumor formation in model of PDAC with liver metastasis. Data represent the mean ± SD, n = 3. Statistical analysis was performed using two‐tailed unpaired student’ s *t*‐test. ^*^, *p* < 0.05; ^**^, *p* < 0.01; ^***^, *p* < 0.001; ^****^, *p* < 0.0001.

Next, STAU2‐overexpressing cell models were established in PANC‐1 and BxPC‐3 cell lines via transfection with either the empty vector (EV) or STAU2‐expressing plasmid constructs (**Figure** **3**A,B). Migration and invasion assay results showed that STAU2 overexpression significantly promoted pancreatic cancer metastasis at cellular levels (Figure 3C,D). The xenograft model based on PANC‐1 cells showed that STAU2 overexpression significantly promoted the proliferation and metastasis of pancreatic cancer cells in vivo. Tumor weight and volume were significantly increased in the STAU2 overexpression group compared to those in the control group (Figure 3E–G), with no significant difference in body weight noted between the two groups (Figure 3H). STAU2 overexpression significantly increased the number of liver nodules and bioluminescence signal intensity in the liver metastasis model mice (Figure 3I,J). Collectively, these results suggested that STAU2 is an important factor in promoting the proliferation and metastasis of tumor cells in vitro and in vivo.

### 2.3 STAU2 is Involved in EMT Pathway Regulation

**Figure 4:**
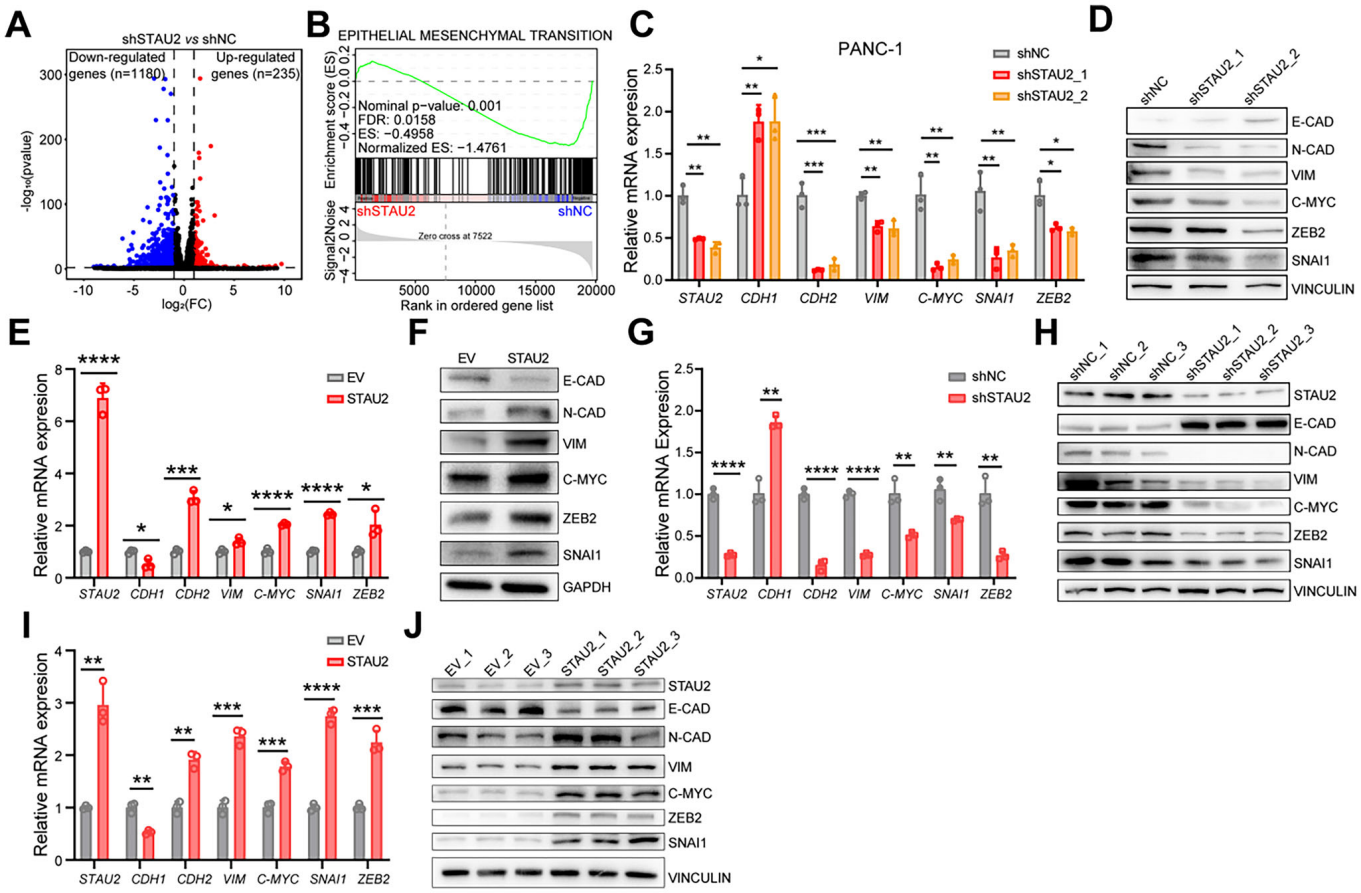
STAU2 is involved in regulating the EMT pathway. A) Volcano plot showing the significant differentially expressed genes between PANC‐1 shNC cells and PANC‐1 shSTAU2 cells. B) GESA analysis revealed that the “HALLMARK_EPITHELIAL_MESENCHYMAL_TRANSITION” is significant down‐regulated in PANC‐1 shSTAU2 cells. C,D) RT‐qPCR (C) and Western blot (D) analysis of STAU2 and EMT pathway marker genes expression in PANC‐1 cells transfected with shNC and two independent shSTAU2 constructs. E,F) RT‐qPCR (E) and Western blot (F) analysis of STAU2 and EMT pathway marker genes expression in PANC‐1 cells that were overexpressed with EV and STAU2. G,H) RT‐qPCR (G) and Western blot (H) analysis of STAU2 and EMT pathway marker genes expression in tumor tissues of shNC and shSTAU2 groups from CDX models. I,J) RT‐qPCR (I) and Western blot (J) analysis of STAU2 and EMT pathway marker genes expression in tumor tissues of EV and STAU2 groups from CDX models. Data represent the mean ± SD, n = 3. Statistical analysis was performed using two‐tailed unpaired student’ s *t*‐test. ^*^, *p* < 0.05; ^**^, *p* < 0.01; ^***^, *p* < 0.001; ^****^, *p* < 0.0001.

The single‐sample gene set enrichment analysis (ssGSEA) was carried out using TCGA database to explore the molecular mechanisms influencing STAU2‐mediated metastasis phenotype. The results showed that STAU2 expression was positively correlated with the EMT pathway markers (Figure S2A, Supporting Information). Subsequently, PDAC patients in TCGA were divided into high‐ and low‐STAU2 groups, and the differentially expressed genes were identified (Figure S2B, Supporting Information). GSEA showed that the HALLMARK_EPITHELIAL_MESENCHYMAL_TRANSITION pathway was significantly enriched in the high‐STAU2 group (Figure S2C, Supporting Information) in clinical samples. Additionally, a volcano plot revealed 1180 down‐regulated and 235 up‐regulated genes in the shSTAU2 group compared to those in the shNC group based on the RNA‐Seq analysis (**Figure** **4**A). GSEA showed that the HALLMARK_EPITHELIAL_MESENCHYMAL_TRANSITION pathway was significantly down‐regulated in the shSTAU2 group (Figure 4B), suggesting that STAU2 knockdown has the potential to inhibit the EMT pathway.

The EMT pathway represents a fundamental cellular morphological transformation, whereby epithelial cells relinquish their polarity and intercellular adhesion, which is indispensable in pathological processes, such as tumor invasion and metastasis.^[^ ^36^ ^]^ EMT is intricately orchestrated by the regulation of transcription factors, predominantly SNAI1, C‐MYC, ZEB1, and ZEB2, cutting the connection between epithelial cell protein (e.g., E‐CAD), increasing interstitial cell marker levels (such as VIM and N‐CAD), and leading to morphological changes in phenotypic characteristics of mesenchymal cells.^[^ ^37^ ^]^ CDH2 (encoding N‐CAD), VIM, ZEB2, SNAI1, and MYC were down‐regulated in PANC‐1 (Figure 4C,D) and BxPC3 cells at both RNA and protein levels (Figure S2D,E, Supporting Information). CDH1 (encoding E‐CAD) expression was up‐regulated after STAU2 knockdown, which indicated that it inhibited the EMT pathway. The opposite trend was observed in STAU2‐overexpressing cells (Figure 4E,F and Figure S2F,G, Supporting Information). RNAs and proteins extracted from tumor tissues of xenograft models validated the above results for STAU2 deletion (Figure 4G,H and Figure S2H,I, Supporting Information) and overexpression (Figure 4I,J) in vivo. These results indicated the critical role of STAU2 in activating the EMT pathway in PDAC and supported the need for further investigation into its regulatory mechanism.

### 2.4 PALLD Promotes the PDAC Progression Acting as a Targeting RNA of STAU2

**Figure 5:**
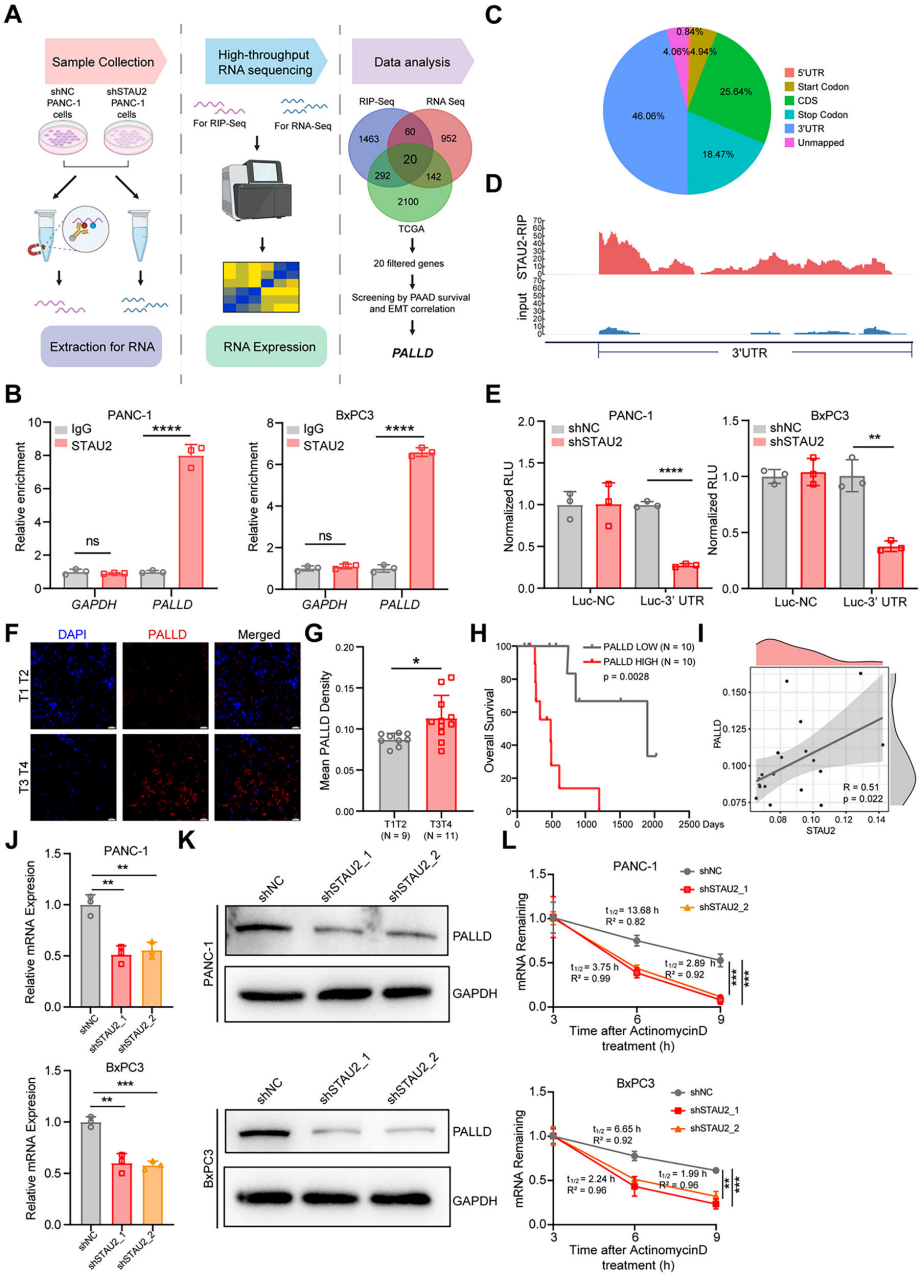
PALLD, a target of STAU2, serves as an important factor in PDAC progression and metastasis. Workflow illustration of STAU2 targeting RNAs that were obtained by RIP sequencing, RNA sequencing and database analysis. B) Relative enrichment of PALLD mRNA in STAU2‐RIP over IgG (control) determined by RT‐qPCR assays in PANC‐1 and BxPC3 cells. GAPDH expression was used as negative control. C) Distribution of STAU2‐enriched RNA peaks across different mRNA regions in PANC‐1 cells detected by RIP‐sequencing. D) Enrichment of RIP‐Seq reads in the 3′ UTR region of PALLD in PANC‐1 cells. E) Relative luciferase activity monitored in shNC and shSTAU2 cells co‐transfected with dual luciferase reporter. Renilla luciferase activity was used for normalization. F) Immunofluorescence staining of tissue microarrays from PDAC patients using PALLD antibody. Scale bar, 20 µm. G) Differential ratio of PALLD mean density in tissue microarray of clinical samples of PDAC at different stages (T_1_T_2_ n = 11, T_3_T_4_ n = 9). H) Overall survival Kaplan‐Meier curve of high (red line, n = 10) and low (blue line, n = 10) PALLD expression PDAC patient samples. Statistical analysis was performed using log‐rank test. I) Correlation analysis of PALLD and STAU2 positive cell ratio in PDAC samples from clinical patient microarray. J) RT‐qPCR analysis of *PALLD* expression in PANC‐1 and BxPC3 cells transfected with shNC and two independent shSTAU2 constructs. K) Western blot analysis of PALLD protein level in PANC‐1 and BxPC3 cells transfected with shNC and two independent shSTAU2 constructs. L) RNA decay curves of PANC‐1 cells and BxPC3 cells transfected with shNC and two independent shSTAU2 constructs treated with actinomycin D. Data represent the mean ± SD, n = 3. Statistical analysis was performed using two‐tailed unpaired student’ s *t*‐test. ^*^, *p* < 0.05; ^**^, *p* < 0.01; ^***^, *p* < 0.001; ^****^, *p* < 0.0001.

STAU2 is an RBP that modulates RNA functions, including translation,^[^ ^11^ ^]^ stability^[^ ^38^ ^]^ and splicing^[^ ^39^ ^]^ by targeting specific downstream motifs. RNA immunoprecipitation sequencing (RIP‐Seq), RNA‐Seq, and public databases were integrated and analyzed to identify the downstream STAU2 RNAs that mediate the cancer‐promoting function in PDAC. The intersection of 20 genes was obtained from 1835 RNAs specifically bound by STAU2 protein in RIP‐Seq, 1174 genes positively correlated with STAU2 in RNA‐Seq, and 2554 genes positively correlated with STAU2 in TCGA‐PAAD (**Figure** **5**A). Different survival models were employed to analyze the intersection of genes in conjunction with the relationship between gene expression and EMT pathway markers, revealing that PALLD is a significant risk factor for PDAC prognosis, exhibiting the highest correlation with the EMT pathway (Figure S3A–C, Supporting Information). Current research studies have shown that PALLD is involved in actin assembly and cytoskeleton stabilization, regulating attachment and breakdown of the extracellular matrix and activating migration and invasion.^[^ ^40^ ^]^ RIP RT‐qPCR results further showed significant and specific STAU2 binding to PALLD RNA (Figure 5B). TCGA data revealed coordinated expression of PALLD with STAU2, implying a potentially critical role of PALLD in PDAC (Figure S3D–F, Supporting Information). To elucidate the molecular mechanism underlying STAU2‐PALLD regulation, RIP‐Seq analysis revealed that STAU2 exhibits preferential binding to the 3′‐untranslated region (UTR) of target RNAs (Figure 5C). Notably, STAU2 exhibited specific enrichment at the PALLD 3′‐UTR (Figure 5D), which was further corroborated by dual‐luciferase reporter assays, validating STAU2's specific binding to this region (Figure 5E).

Furthermore, microarray immunofluorescence staining assay with clinical PDAC samples revealed that patients in advanced PDAC stages (T_3 – 4_) exhibited a notably elevated positive ratio of PALLD compared to those in earlier stages (T_1 – 2_), suggesting the presence of PALLD up‐regulation in clinical progression of PDAC (Figure 5F,G). The subsequent survival analysis of 20 clinical samples that were categorized into the high‐ and low‐PALLD groups indicated that the survival rate in the high‐PALLD group was significantly lower than that in the low‐PALLD group (Figure 5H). Notably, the correlation between STAU2 and PALLD (Figure 5I) highlighted the synchronous expression of STAU2 and PALLD in patient samples (p = 0.022). Moreover, the consistent trend in PALLD expression at both RNA and protein levels with STAU2 was also confirmed in PANC‐1 and BXPC‐3 cells with STAU2 knockdown or overexpression (Figure 5J,K and Figure S3G,H, Supporting Information). The RNA stability assay results showed that PALLD mRNA levels were significantly reduced in PANC‐1 and BxPC3 cells with STAU2 knockdown within 9 h (Figure 5L). In summary, PALLD acts as a critical risk factor in PDAC and is a down‐stream STAU2 target that is stabilized by STAU2.

### 2.5 STAU2‐PALLD Axis Regulates PDAC Cell Invasion and Migration via the EMT Pathway

**Figure 6:**
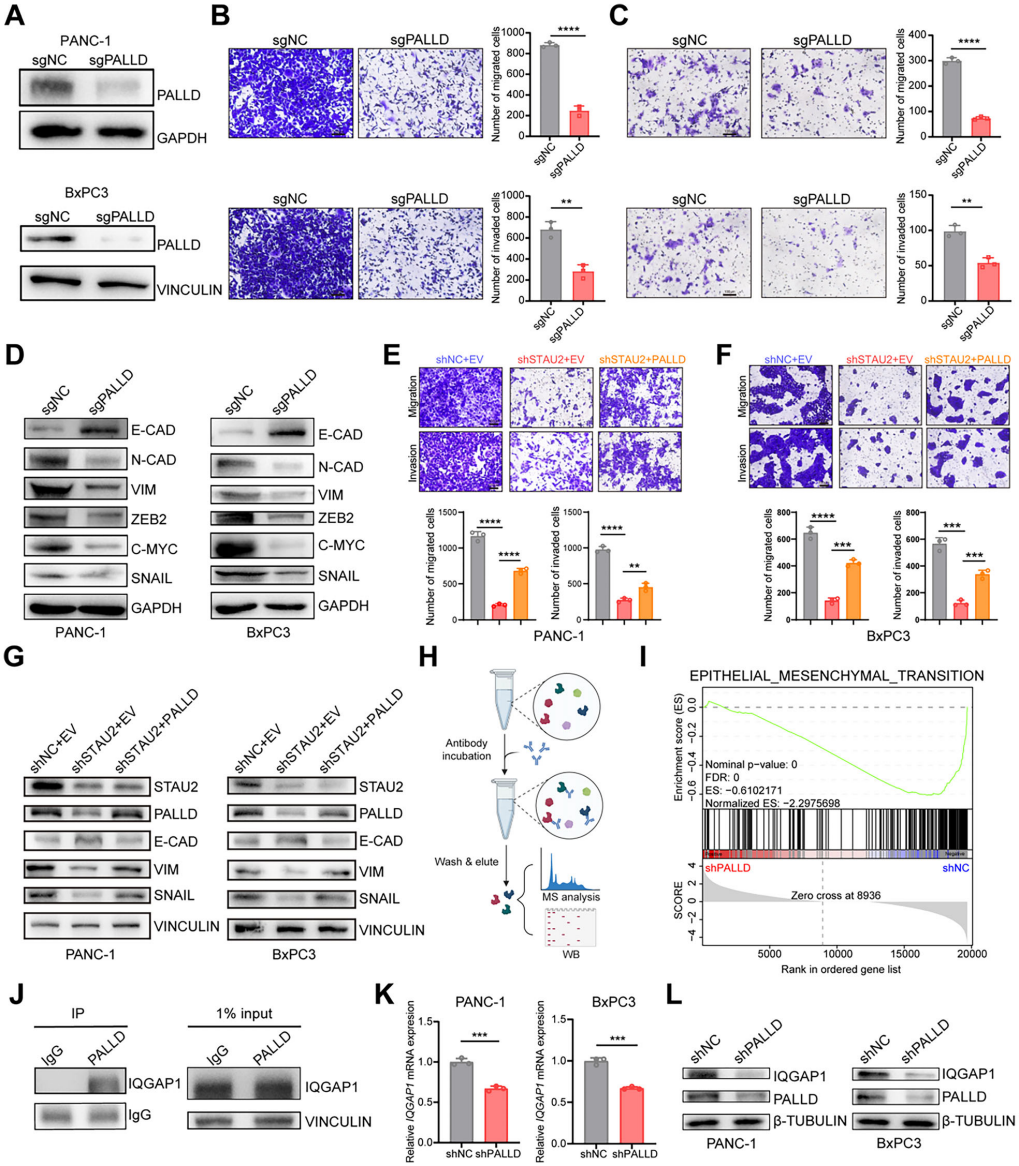
PALLD mediates IQGAP1 to regulate PDAC cell invasion and migration through the EMT pathway. A) Western blot analysis of PALLD protein levels in PANC‐1 cells and BxPC3 cells transfected with sgNC and sgPALLD constructs. B,C) Migration and Invasion ability of PANC‐1 (B) and BxPC3 (C) cells with PALLD knockout or control. Representative images of migrated and invaded cells were shown. Scale bar, 100 µm. D) Western blot analysis of marker protein of EMT pathway in PANC‐1 cells and BxPC3 cells transfected with sgNC and sgPALLD constructs. E,F) Representative images and quantification of migrated and invaded cells of control (shNC + EV), shSTAU2, and shSTAU2 + PALLD (vector with PALLD overexpression) PANC‐1 and BxPC3 cells. Scale bar, 100 µm. G) Western blot analysis of marker protein of EMT pathway in PANC‐1 cells and BxPC3 cells that transfected with control (shNC + EV), shSTAU2, and shSTAU2 + PALLD constructs. H) IP‐MS schema diagram. I) PALLD silencing significantly inhibited the EMT pathway by GSEA analysis. J) Specific binding of IQGAP1 to PALLD as demonstrated by western blot. K,L) The reduction of IQGAP1 RNA and protein levels in shPALLD PANC‐1 and BXPC3 cells confirmed by RT‐qPCR and western blot. Data represent the mean ± SD, n = 3. Statistical analysis was performed using two‐tailed unpaired student’ s *t*‐test. (^*^, *p* < 0.05; ^**^, *p* < 0.01; ^***^, *p* < 0.001; ^****^, *p* < 0.0001).

CRISPR‐Cas9 was utilized to knockdown PALLD in PANC‐1 and BxPC3 cells in order to investigate whether STAU2 regulation of metastasis in PDAC was mediated by PALLD (**Figure** **6**A). Transwell migration and invasion assays demonstrated that PALLD knockdown significantly reduced the migratory and invasion capabilities of PDAC cells (Figure 6B,C), suggesting that PALLD played an important role in PDAC metastasis. Furthermore, PALLD knockdown led to a decreased expression of key proteins which involved in the EMT pathway (Figure 6D). Then, PDAC cells with both PALLD overexpression (Figure S3I, Supporting Information) and STAU2 knockdown were generated for rescue experiments to explore how the regulatory relationship between STAU2 and PALLD affects migration and invasion. The results demonstrated that PALLD overexpression partially rescued the reduction in cell migration and invasion induced by STAU2 knockdown, implying a regulatory role for PALLD in STAU2‐mediated PDAC cell metastasis (Figure 6E,F). Collectively, the results indicated that STAU2 regulated PDAC metastasis via the PALLD‐mediated EMT pathway (Figure 6G). Collectively, the results indicated that STAU2 regulated PDAC metastasis through PALLD mediated EMT pathway. However, the underlying mechanisms by which the STAU2‐PALLD axis promotes the hyperactivation of the EMT pathway in PDAC remain to be elucidated.

Immunoprecipitation‐Mass Spectrometry (IP‐MS) (Figure 6H) and RNA‐Seq (Figure 6I) were used to explore the mechanisms of the STAU2‐PALLD axis‐mediated EMT. IP‐MS of PALLD identified 406 proteins that specifically bound to PALLD, while PALLD knockdown cell line RNA‐Seq results identified 1552 significantly down‐regulated genes, with 30 intersecting key genes (Figure S4A, Supporting Information). The GEPIA2 online tool employed to analyze the OS and recurrence‐free survival (RFS) of 30 genes revealed four genes, including *CAV1, TMEM43*, *IQGAP1*, and *CDK1*, that were significantly correlated with pancreatic cancer risk based on their differential expression between tumor and normal tissues (Figure S4C,D, Supporting Information). Combined with the top protein results for PALLD‐specific binding (Figure S4B, Supporting Information), IQGAP1 was found to be a PALLD down‐stream. IQGAP1 was reported as a regulator of multiple GTPases directly binding to the down‐stream proteins. Meng‐Ru Shen et al. reported that Rac1 is a core member of the Rho family, which is the most important class of small GTPases that interact with IQGAP1, was activated by IQGAP1, thereby driving the EMT process and promoting tumor invasion and metastasis.^[^ ^41^ ^]^ The present ssGSEA derived from TCGA supported this conclusion (Figure S4E, Supporting Information). Additionally, we proved that IQGAP1 was specifically bound to PALLD (Figure 6J) and confirmed reduced IQGAP1 RNA and protein levels in shPALLD PANC‐1 and BXPC3 cells (Figure 6K,L). The above results suggested that the STAU2‐PALLD axis mediates IQGAP1 to regulate the invasion and migration of PDAC cells via the EMT pathway.

### 2.6 STAU2‐ASO Inhibits PDAC Cell Progression, Invasion, and Migration by Regulating the EMT Pathway In Vitro

**Figure 7:**
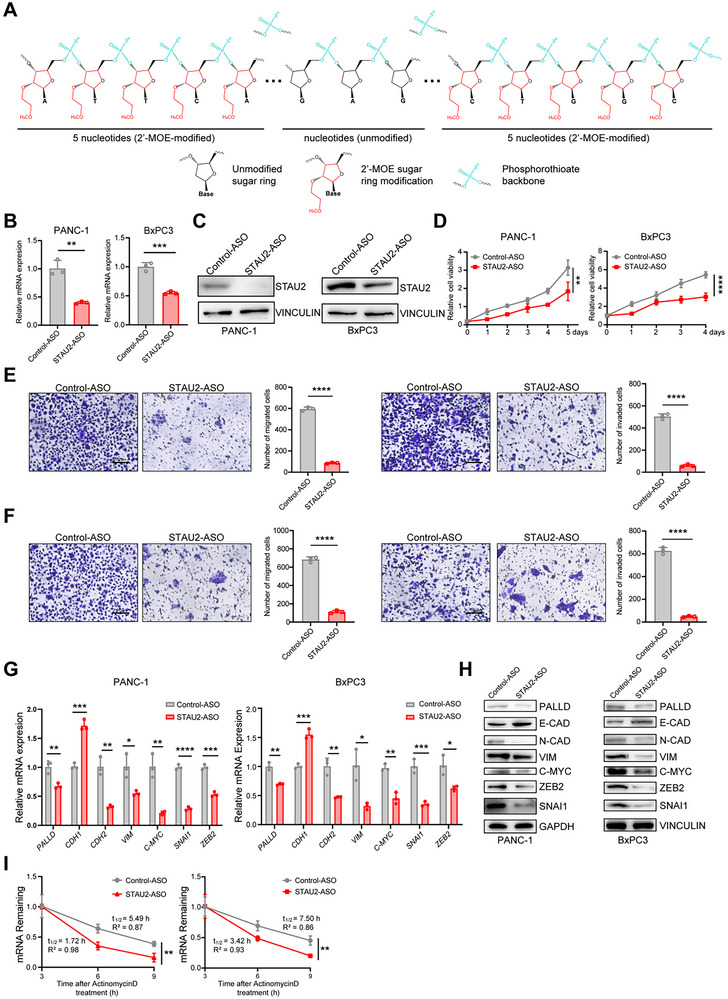
STAU2‐ASO inhibits the progression and metastasis of PDAC by regulating EMT pathway in vitro. A) Sequence and modification design of STAU2‐ASO. B) RT‐qPCR analysis of *STAU2* expression in PANC‐1 cells and BxPC3 cells treated with Control‐ASO and STAU2‐ASO. C) Western blot analysis of STAU2 protein levels in PANC‐1 cells and BxPC3 cells treated with Control‐ASO and STAU2‐ASO. D) Cell viability of PANC‐1 cells and BxPC3 cells treated with Control‐ASO and STAU2‐ASO during a 5‐day course. E) Migration and invasion ability of PANC‐1 cells treated with Control‐ASO and STAU2‐ASO. Scale bar, 100 µm. F) Migration and invasion ability of BxPC3 cells treated with Control‐ASO and STAU2‐ASO. Representative images of migrated and invaded cells were shown. Scale bar, 100 µm. G) RT‐qPCR analysis of *PALLD* and EMT pathway marker genes expression in PANC‐1 cells and BxPC3 cells treated with Control‐ASO and STAU2‐ASO. H) Western blot analysis of marker protein of EMT pathway in PANC‐1 cells and BxPC3 cells treated with Control‐ASO and STAU2‐ASO. I) RNA decay curves of PANC‐1 and BxPC3 cells treated with Control‐ASO and STAU2‐ASO. Data represent the mean ± SD, n = 3. Statistical analysis was performed using two‐tailed unpaired student’ s *t*‐test. ^*^, *p* < 0.05; ^**^, *p* < 0.01; ^***^, *p* < 0.001; ^****^, *p* < 0.0001.

The tumor‐promoting role of STAU2 in PDAC highlights its potential as a novel therapeutic target. However, the lack of full‐length STAU2 structure and the absence of a druggable RBP pocket pose significant challenges to the development of small‐molecule inhibitors directly targeting STAU2 protein. Recently, more and more attention has been paid to the development of ASO, which offer several advantages over traditional drugs, such as enhanced targeting specificity and prolonged efficacy.^[^ ^42^, ^43^, ^44^ ^]^ We developed STAU2‐ASO with 2′‐MOE terminal five bases and phosphorothioate backbone modification (**Figure** **7**A), which significantly suppressed the STAU2 level of both at the mRNA and protein levels (Figure 7B,C). Notably, compared with Control‐ASO, the treatment with STAU2‐ASO significantly inhibited the proliferation, invasion, and migration of PDAC cells (Figure 7D–F). Furthermore, STAU2‐ASO down‐regulated the EMT‐related molecules both at the RNA and protein levels (Figure 7G,H). RNA stability assay showed that PALLD mRNA was significantly reduced in PANC‐1 and BxPC3 cells treated with STAU2‐ASO (Figure 7I). In summary, STAU2‐ASO exhibited potent anti‐tumor activity and significantly inhibited the proliferation and metastasis of pancreatic cancer cells in vitro.

### 2.7 STAU2‐ASO Inhibits PDAC Progression and Metastasis by Regulating the EMT Pathway In Vivo

**Figure 8:**
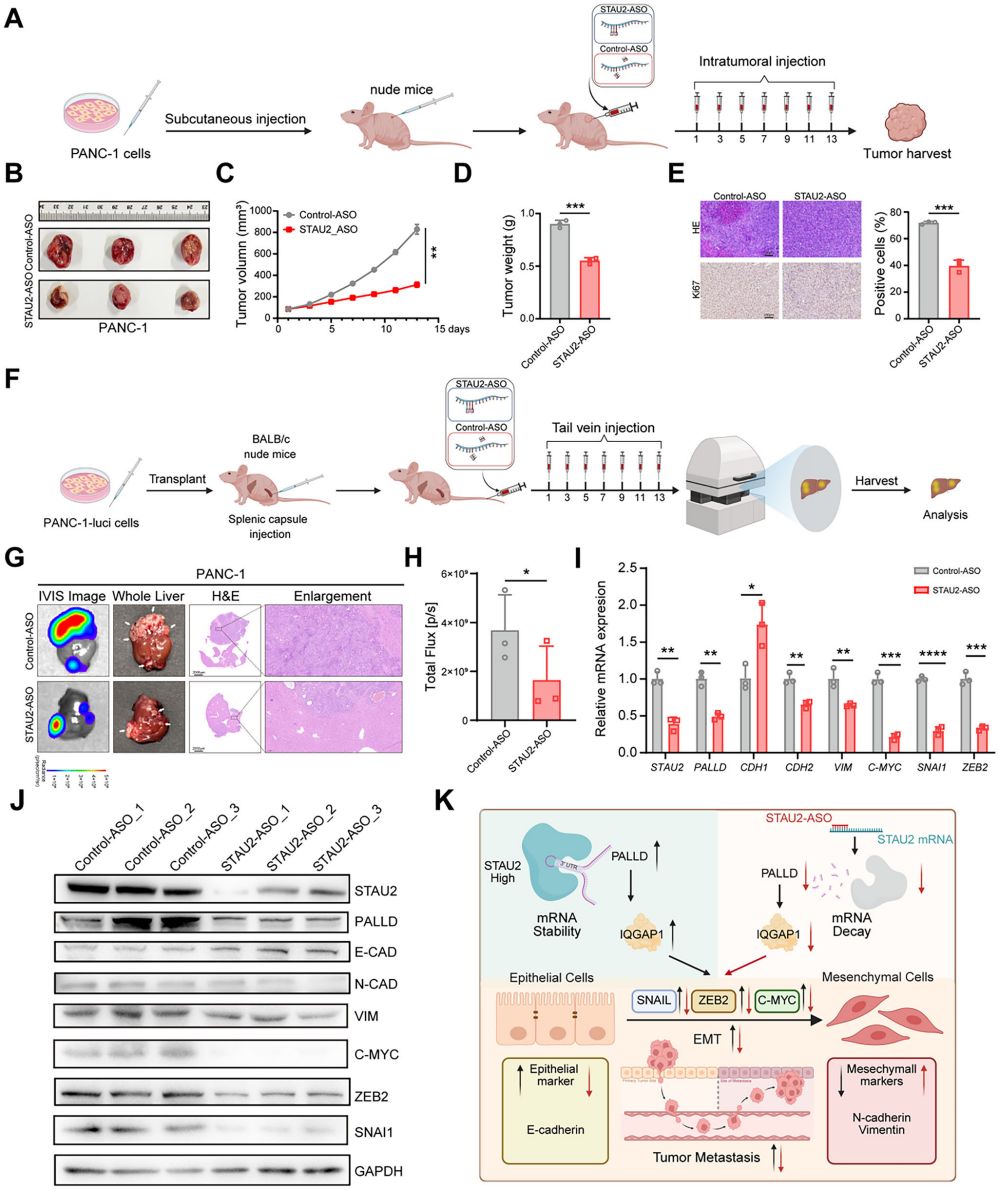
STAU2‐ASO inhibits the progression and metastasis of PDAC by regulating EMT pathway in vivo. A) Overview of STAU2‐ASO anti‐tumor growth assays in vivo. B–D) BALB/c nude mice subcutaneously transplanted with PANC‐1 cells were treated with Control‐ASO or STAU2‐ASO. Tumor volumes were measured every 2 days (C), and after mice were euthanized, tumors were excised, photographed (B), and weight (D). E) Quantitative analysis of the ratio of Ki67 positive cells in three independent tumors from the STAU2‐ASO treatment group and the Control‐ASO treatment group. Scale bar, 100 µm. F) Overview of the protocol for STAU2‐ASO anti‐tumor metastasis assay in vivo. G) Representative bioluminescent images (G, column of IVIS image), photographs (G, column of whole liver, arrows point to the tumor nodules) and HE staining with enlargement (G, columns of HE and enlargement) are shown, respectively. Scale bar, 2000 µm (H&E); 100 µm (enlargement). H) Quantified after tumor formation in model of PDAC with liver metastasis. I) RT‐qPCR analysis of STAU2 and evaluation of the expression of EMT pathway marker genes in tumor tissues of xenograft models treated with Control‐ASO or STAU2‐ASO. J) Western blot analysis of EMT pathway marker proteins in a liver metastasis PDAC model treated with Control‐ASO or STAU2‐ASO. K) Working model of STAU2 regulating PDAC metastasis and the mechanism of action of STAU2‐ASO. Data represent the mean ± SD, n = 3. Statistical analysis was performed using two‐tailed unpaired student’ s *t*‐test. ^*^, *p* < 0.05; ^**^, *p* < 0.01; ^***^, *p* < 0.001; ^****^, *p* < 0.0001.

A PANC‐1 xenograft model was established (**Figure** **8**A) to evaluate the in vivo anti‐tumor efficacy of STAU2‐ASO. STAU2‐ASO (10 nmol L^−1^) or Control‐ASO was administered every other day post‐modeling. The STAU2‐ASO group exhibited a significantly reduced tumor volume of ≈300 mm^3^ compared to that in the Control‐ASO group, which reached ≈900 mm^3^ on day 13 (Figure 8B,C). Similarly, treatment with STAU2‐ASO led to a reduction in tumor weight by more than half (Figure 8D), with a tumor inhibition rate of 62.1%. Importantly, STAU2‐ASO administration did not result in a statistically significant decrease in body weight throughout the treatment period (Figure S5A, Supporting Information). The Ki67 staining demonstrated significant down‐regulation in tumors treated with STAU2‐ASO compared to the controls, indicating a substantial reduction in tumor cell proliferation in vivo (Figure 8E). HE staining of organs, including the heart, liver, spleen, lung, and kidney, revealed well‐preserved tissue in the STAU2‐ASO group without obvious necrosis or inflammation (Figure S5B, Supporting Information). These results elucidated the favorable antitumor efficacy and safety profile of STAU2‐ASO in vivo. Similar results were observed in the BxPC3 xenograft model with a tumor suppression rate of 44.2% (Figure S5C–H, Supporting Information).

A tumor metastasis model was established via an intrasplenic injection of PANC‐1 cells to investigate the efficacy of STAU2‐ASO against PDAC metastasis in vivo. STAU2‐ASO (10 nmol L^−1^) or Control‐ASO was administered on days 1, 3, 5, 7, 9, 11, and 13 after model establishment (Figure 8F). Photographs of liver tissue and signal intensity showed that STAU2‐ASO significantly inhibited pancreatic cancer liver metastasis and decreased the number of liver tumor nodules on day 14 (Figure 8G,H). In addition, STAU2‐ASO treatment led to a marked STAU2 expression down‐regulation and the EMT pathway suppression in tumor tissues, confirming its potent anti‐metastatic efficacy in PDAC (Figure 8I,J). Similar results were observed in the BxPC3 xenograft model (Figure S5I,J, Supporting Information). STAU2 expression and the EMT pathway were significantly inhibited in the BxPC3 xenograft model (Figure S5K,L, Supporting Information). In summary, our work revealed and comprehensively validated the promoting role of STAU2‐PALLD axis in the progression and metastasis of pancreatic cancer (Figure 8K), suggesting that STAU2 as a novel drug target for therapeutic intervention. Moreover, STAU2‐ASO was developed as the first inhibitor targeting STAU2, demonstrating anti‐tumor activity both in vitro and in vivo and providing a promising novel therapeutic strategy for pancreatic cancer treatment.

## 3 Discussion

PDAC is a highly aggressive cancer that poses significant challenges in diagnosis and treatment, resulting in late detection and poor survival rates. Advanced PDAC is often characterized by tumor metastasis, in which EMT plays a pivotal role in promoting metastatic behavior by endowing cancer cells with migratory and invasive properties.^[^ ^45^, ^46^ ^]^ This critical involvement of EMT in PDAC progression underscores the urgent need to develop novel therapeutic strategies targeting the EMT regulatory pathways in order to improve clinical outcomes. Recent studies utilizing genome‐wide in vivo CRISPR screening have identified STAU2 as a crucial molecule in myeloid leukemia.^[^ ^47^ ^]^ STAU2 knockout significantly inhibited the myeloid leukemia progression. The important roles of STAU2 in myeloid leukemia support its potential significance in cancer therapy.

The correlation between STAU2 expression and EMT was investigated in the present study. STAU2 overexpression promoted the EMT process in PDAC, which was further confirmed by knockdown and overexpression experiments at both cellular and animal model levels. Previous studies have discovered a mechanism between EMT in PDAC and the regulation of transcriptional factors, such as ZEB2, c‐MYC, and SNAI1. Consistently, altered STAU2 expression resulted in ZEB2, c‐MYC, and SNAI1 expression changes and regulated EMT‐induced PDAC cell migration‐related phenotypes. The present findings highlight STAU2 as a pivotal molecule in EMT‐mediated metastasis in pancreatic cancer.

Mechanistically, PALLD was identified as a downstream STAU2 target using RIP‐seq, RNA‐seq, and bioinformatics analyses. Dual‐luciferase reporter assays and RNA stability experiments confirmed that STAU2 specifically binds to the 3′UTR of *PALLD* and stabilizes its mRNA. Rescue experiments overexpressing PALLD in shSTAU2 cell lines validated the regulatory role of the STAU2‐PALLD‐EMT axis in pancreatic cancer cell migration, invasion, and EMT pathway expression. Further IP‐MS analyses revealed that STAU2‐PALLD modulates EMT via IQGAP1 regulation. Collectively, these findings revealed that the STAU2‐PALLD axis regulates IQGAP1, promoting PDAC metastasis via EMT activation.

The lack of druggable pockets on RBPs has hindered the discovery of STAU2‐targeted inhibitors despite the reports on the role of STAU2 in tumorigenesis. Leveraging the RNA sequence of STAU2, a stable, efficient, and safe ASO was developed for the treatment of PDAC. STAU2‐ASO exhibited potent degradation activity against STAU2 mRNA in vitro and effectively inhibited the proliferation and metastasis of PDAC cells. More importantly, STAU2‐ASO demonstrated significant anti‐tumor activity in multiple CDX models with a favorable safety profile, suggesting its potential for further evaluation as a novel drug for PDAC therapy.

In summary, our study demonstrated that the STAU2‐PALLD axis promoted the progression and metastasis of pancreatic cancer, and developed STAU2‐ASO as the first inhibitor targeting STAU2‐PALLD axis. Given the oncogenic role of STAU2 and the potential of ASO to minimize molecular off‐target effects,^[^ ^48^ ^]^ STAU2‐ASO represents a promising therapeutic option for patients with pancreatic cancer and potentially other cancer types in the foreseeable future.

## 4 Experimental Section

### The Public Database Data Collection and Analysis

TGCA (The Cancer Genome Atlas, https://portal.gdc.cancer.gov/)

GDC (Genomic Data Commons, https://portal.gdc.cancer.gov/)

GTEx (Genotype‐Tissue Expression, https://commonfund.nih.gov/gtex)

UCSC Xena (https://xena.ucsc.edu/)

GEO (https://www.ncbi.nlm.nih.gov/geo/). Data with accession number GSE162791 and GSE154778 in GEO was used for analysis.

GSEA (Gene Set Enrichment Analysis, (www.gsea‐msigdb.org/gsea/)

Kaplan‐Meier Plotter (https://kmplot.com/)

### Clinical Samples

With the informed consent of the patients, 20 PDAC tumor tissues were obtained from Huai'an First People's Hospital for sectioning and immunofluorescence staining. All human samples used in this study were identified and approved by the Huai'an First People's Hospital medical ethics committee. Clinicopathologic information of each patient was listed in Table S1 (Supporting Information).

### Immunofluorescence

Sections were fixed with 4% paraformaldehyde and then incubated with 0.5% Triton X‐100 and 5% bovine serum albumin (BSA). After being incubated with the first antibody at 4 °C overnight and following the secondary antibody, the cells were stained with DAPI, STAU2 (GTX115836, GeneTex), PALLD (10853‐1‐AP, Proteintech) and visualized with a fluorescence microscope.

### Cell Culture

The PDAC cell lines PANC‐1 and BxPC3, and normal pancreatic cell line HPDE6‐C7, were all provided by the American Type Culture Collection (ATCC). The PANC‐1 and HPDE6‐C7 cell lines were cultured in DMEM medium supplemented with 10% endotoxin‐free fetal bovine serum (FBS) (10270‐106, Gibco), while the BxPC3 cell line was maintained in RPMI 1640 medium containing 10% endotoxin‐FBS. 1% penicillin‐streptomycin was added to the media. All cell lines used in this study were cultured in the recommended culture medium at 37 °C in 5% CO_2_. All the cell lines were authenticated via STR identification.

### Cell Proliferation Assays

Cell proliferation was quantitatively assessed utilizing the Cell Counting Kit‐8 (CCK‐8, Share‐bio). 5000 cells per well were seeded into 96‐well plates and incubated for 5 days. Daily, CCK‐8 reagent was dispensed into each well, followed by a 4‐h incubation in a controlled environment. Absorbance measurements at 450 nm were conducted using a microplate reader (Bio‐Tek Synergy H1, Vineland, NJ, USA) integrated with an enzyme plate analyzer. To evaluate cell growth kinetics, cell viability was determined at multiple time points (0, 24, 48, 72, and 96 h) across three independent experimental replicates.

### Migration Assays

Cell migration was examined in vitro utilizing the transwell chamber system (Falcon, 353 097). Initially, 500 µL of medium containing 10% FBS was added to the lower chamber at room temperature, while 200 µL of basal medium without FBS containing 2 × 10^4^ cells was dispensed into the upper chamber of the transwell. After 24 h of incubation within the transwell chamber, migrated cells were fixed to the bottom of the chamber using 4% paraformaldehyde and stained with 0.1% crystal violet. Non‐migrated cells on the upper surface were then removed. The number of migrated cells on the lower surface was counted under a microscope to determine the migration capacity.

### Invasion Assays

The day before the experiment, the upper surface of the membrane at the bottom of the transwell chamber was coated with a 1:8 dilution of 50 mg/L matrigel and allowed to dry. The remaining steps were identical to the migration experiment.

### Lentiviral Production and Cellular Infection

Lentiviral particles harboring shRNA targeting STAU2 (shSTAU2), shRNA targeting PALLD (shPALLD), non‐targeting scrambled control (shNC), empty vector controls (EV) as negative counterparts, and overexpression plasmids targeting both STAU2 or PALLD were custom‐designed and procured from Genechem Co. Ltd. (Shanghai, China). Similarly, lentiviral constructs encoding Cas9 with a single guide RNA (sgRNA) against PALLD (sgPALLD) and its respective scrambled control (sgNC) were constructed and obtained from Tsingke Biotechnology Co. Ltd. (Beijing, China).

A total of 5 × 10^4^ cells were plated into individual wells of a 6‐well plate and incubated for 24 h to allow for proper cell adherence. Subsequently, the cells were transduced with the respective lentiviral particles in the presence of HitransG P transfection enhancer (Genechem, REVG005), according to the manufacturer's protocol. After 16–24 h of transduction, the medium containing lentiviral particles was aspirated, and the cells were allowed to recover and proliferate for an additional 3–5 days. Throughout this period, GFP fluorescence was monitored daily as an indicator of successful transduction. To enrich for successfully transduced cells, a 24‐h treatment with 2 µg mL^−1^ of puromycin was implemented. This step effectively eliminated non‐transduced cells, ensuring a high purity of transduced cell populations. Following selection, all transduced cells were rigorously validated through RT‐qPCR and western blotting to confirm target gene modulation at both the mRNA and protein levels. To maintain the transduced phenotype, cells were continuously cultured in a medium supplemented with 1 µg mL^−1^ of puromycin. The lentiviral particles were employed at a multiplicity of infection (MOI) of 10, ensuring efficient transduction rates while minimizing potential cytotoxic effects. The detailed nucleotide sequences of shSTAU2, shNC, shPALLD, sgPALLD, sgNC, and primer sequences for overexpression plasmids are available in the Table S2 (Supporting Information).

### Real‐Time Quantitative PCR (RT‐qPCR)

The specific methods of RT‐qPCR were similar to those in our previous article.^[^ ^49^, ^50^ ^]^ Total RNA was isolated from cells employing the RNA‐easy reagent (Vazyme; Catalog No. R701‐01). Subsequently, the extracted RNA was reverse transcribed into cDNA using the Hiscript III 1st Strand cDNA Synthesis Kit (Vazyme; Catalog No. R323‐01). The quantitative assessment of gene expression was conducted via RT‐qPCR, leveraging the ChamQ SYBR qPCR Master Mix (Vazyme; Catalog No. Q331‐02). The assembled RT‐qPCR reactions were then subjected to amplification and detection on an ABI 7500 Fast Real‐Time PCR System (Applied Biosystems, USA). To ensure statistical robustness, each sample was analyzed in triplicate, with technical replicates providing a robust basis for data interpretation. For normalization purposes, GAPDH was selected as the reference gene, owing to its consistent expression across various cellular conditions. Relative gene expression levels were subsequently calculated utilizing the 2^−ΔΔCt^ method. Specific information on primers is provided in the Table S2 (Supporting Information).

### Western Blot

The specific methods of western blot were similar to those in our previous article.^[^ ^51^ ^]^ Cell lysates were prepared using RIPA buffer (Thermo Fisher Scientific; catalog no. 89 901) supplemented with phosphatase (Roche, Basel; catalog no. 0 490 684 5001) and protease inhibitors (Roche; catalog no. 0 469 313 2001). Protein quantification was achieved via Bradford assay (Beyotime; catalog no. P0006), followed by denaturation in SDS sample buffer and electrophoresis on SDS‐PAGE gels. Resultant proteins were transferred onto PVDF membranes. Membrane blocking, primary antibody incubation (diluted in blocking solution targeting specific proteins), and subsequent washes were conducted in a buffer comprising 0.05% (v/v) Tween‐20 and 5% (w/v) nonfat dry milk, with all incubations at 4 °C overnight. Following four washes with blocking buffer, membranes were incubated with horseradish peroxidase‐conjugated secondary antibodies. Specific information on antibodies are provided in the Table S3 (Supporting Information).

### Immunoprecipitation‐Mass Spectrometry (IP‐MS)

Cells were lysed with a buffer and kept on ice for 5 min. After thorough mixing, they were centrifuged at 13000 g for 10 min to release nuclear proteins. Subsequently, 10 µL of anti‐PALLD antibody and rabbit IgG were added to the proteins and incubated for 2 h at 4 °C. These proteins were then co‐incubated overnight at 4 °C with 2% BSA, antibodies, and 10 mg of protein A‐Sepharose beads. Following this, the proteins were eluted three times and fractionated using SDS‐PAGE. The gel pieces were incubated in the dark at room temperature for 45 min, washed with 50 mM NH_4_HCO_3_, and dehydrated using acetonitrile. They were resuspended in NH_4_HCO_3_ and trypsin on ice for 1 h, then trypsin digestion was conducted overnight at 37 °C after removing excess liquid. Peptides were extracted using a mixture of acetonitrile and formic acid, dried, and resuspended in a solution of acetonitrile and formic acid. The tryptic peptides were dissolved in solvent A and loaded onto an in‐house reversed‐phase column. MS/MS analysis was performed using Q ExactiveTM Plus coupled to UPLC, following NSI source application. The scan range was 350 to 1800 m/z, and intact peptides were detected at a resolution of 70000 in the Orbitrap. Selected peptides underwent MS/MS analysis, and the data were processed using Proteome Discoverer 1.3.

### RNA‐Immunoprecipitation

Cell lysis was performed using a tailored lysis buffer (composition: 100 mM KCl, 5 mM MgCl_2_, 10 mM Hepes pH 7.0, 1 mm DTT, 50 units mL^−1^ RNase inhibitor, 1× protease inhibitor cocktail, 1× PBS) and the mixture was incubated at 4 °C for 2 h to ensure complete cellular disruption. Subsequently, 10% of the lysate was designated as the input control. Protein A‐Agarose beads (Sigma‐Aldrich, P1406‐250MG, Darmstadt, Germany) were preconditioned by sequential washes with PBS (three times) followed by a 30‐min incubation in 2% BSA at 4 °C. These beads were then incubated with either 10 µL of STAU2‐specific antibody or IgG (as a negative control) at 4 °C for 2 h to form the bead‐antibody complex. The bead‐antibody complexes were subsequently combined with the cell lysate and incubated overnight at 4 °C to facilitate RNA‐protein interaction capture. Following incubation, the beads were thoroughly washed with PBS to remove unbound material, and the bound RNA was isolated. The purified RNA was subjected to downstream analyses, including reverse transcription quantitative polymerase chain reaction and RIP‐Seq, to characterize the associated RNA transcripts (Guangzhou Genedenovo Biotechnology Co., Ltd).

### RNA‐Seq and Data Analysis

Cells were collected and subjected to total RNA extraction employing the Beyozol Total RNA Extraction Reagent (Beyotime, R0011). Subsequently, total mRNA was enriched utilizing Obligo(dT) beads, followed by fragmentation into short segments and reverse transcription into cDNA with random primers. After synthesis of the second‐strand cDNA, the fragments underwent rigorous purification, end‐repair, poly(A) tailing, and ligation to Illumina sequencing adapters. Size selection of the ligation products was conducted through agarose gel electrophoresis, ensuring appropriate fragment sizes for sequencing. The resulting products were then PCR‐amplified and subjected to high‐throughput sequencing on the Illumina HiSeq2500 platform by Genedenovo Biotechnology Co., Ltd (Guangzhou, China). Gene expression levels were quantified using the FPKM metric, providing a comprehensive view of transcriptional activity. Differential expression analysis was performed between two distinct groups utilizing DESeq2 software, with a stringent criterion of a false discovery rate (FDR) < 0.05 and an absolute fold change ≥ 2 employed to identify significantly differentially expressed genes. Bioinformatic interrogation of the sequencing data was facilitated by the Omicsmart platform (https://www.omicsmart.com), enabling in‐depth analysis and interpretation of the molecular signatures under investigation.

### RNA Stability Assay

To detect RNA stability, Actinomycin D, which inhibits the synthesis of nascent RNA, was added to a six‐well plate containing 3×10^5^ cells per well that were seeded the previous day. The concentration of Actinomycin D in each well was adjusted to 10 µg mL^−1^. Cells were collected at 3, 6, and 9 h after the addition of Actinomycin D, and RNA was extracted. Subsequently, the level of PALLD mRNA in the cells was detected using the RT‐qPCR.

### Dual‐Luciferase Reporter Assay

The sequence of *PALLD* 3′ UTR or control was cloned into GV272 vector (containing firefly luciferase gene) to construct Luc‐3′ UTR and Luc‐NC luciferase vectors. Luc‐3′ UTR or Luc‐NC plasmids were co‐transfected into cells with renilla luciferase plasmid using transfection reagent Lipo8000. Plasmids were packaged and constructed by Genechem Co., LTD. (Shanghai, China). Luciferase activity was measured 2 days after transfection using a dual luciferase reporter kit (Vazyme) in a microplate reader (Bio‐Tek, SynergyH1). Firefly luciferase activity was normalized to renilla luciferase activity.

### ASO Design, Selection, and Transfection

The antisense oligonucleotide STAU2‐ASO targeting STAU2 with the sequence 5′ ATTCATAAGGAGTTCCCTGGC 3′ and the scrambled control were constructed and synthesized by SYNBIO technologies Inc (Suzhou, China). STAU2‐ASO and Control‐ASO were modified with phosphorothioate and 2′‐MOE modification at both ends. These modifications were used to resist nuclease degradation, enhance the binding affinity of ASO to complementary RNA, and reduce immune stimulation and adverse reactions to a certain extent. 4 × 10^5^ cells were seeded onto a 6‐well plate, and ensure that the cell confluence reaches 70%‐80% before transfection. Use lipo8000 transfection reagent (Beyotime, C0533) to perform cellular transfection of ASO. After 24 to 48 h of transfection, verify its expression using Western blotting. The detailed nucleotide sequences of ASO are available in the Table S2 (Supporting Information).

### Animal Studies

Mice were kept at a temperature of 25 °C, relative humidity of ≈60 to 70%, and a light and dark time of 12 h. Tumor volume and body weight were measured every 2 days. After the experiment, euthanasia in mice, and the tumor and dissect and photographed, and weighing vital organs. Tumors and organs were stored in 10% formaldehyde solution or directly for further analysis. All animal experiments were performed in accordance with the guidelines for laboratory animals of China Pharmaceutical University.

### PDAC Cell Line Xenograft Mouse Model

4‐5‐week‐old female BALB/c‐nude mice were purchased from Shanghai SLAC Laboratory Animals Co. Ltd. shNC PANC‐1 and shSTAU2 PANC‐1 cells in active growth phase were collected and prepared into cell suspension under sterile conditions. 0.1 ml of cell suspension was injected into the armpit of mice, with ≈5 × 10^6^ cells per mouse. The diameter of the transplanted tumor was measured by the vernier caliper. After the tumor grew to 80–100 mm^3^, the animals were randomly divided into two groups, and the anti‐tumor effect of the tested drugs was observed by measuring the tumor diameter.

### Liver Metastasis Model

Six‐week‐old BALB/c‐nude female mice were purchased from Shanghai SLAC Laboratory Animals Co. Ltd. Cell suspensions of PANC‐1‐luci and BxPC3‐luci with a concentration of 5×10^7^ cells mL^−1^ was collected and placed in ice box for later use. Mice were anesthetized through intraperitoneal injection of 10% chloral hydrate and positioned on their sides, securely fixed on a surgical board to fully expose their spleens. A small incision was made in the skin over the spleen using ophthalmic surgical scissors, the skin was stretched open, and the spleen was gently pulled out with forceps. A 1 mL insulin syringe was used to aspirate the cell suspension, and 50 µL of it was slowly injected into the splenic capsule. After injection, the spleen was returned to the abdominal cavity, the skin was sutured, and the area was disinfected with iodine. Serial live imaging was conducted weekly post in situ cell injection. Mice were euthanized after the third imaging session, followed by liver procurement and ex vivo fluorescence microscopy of tissue sections.

### STAU2‐ASO Efficacy Evaluation In Vivo

After the CDX model of PANC‐1 and BxPC3 cells were established, the efficacy of STAU2‐ASO was evaluated. STAU2‐ASO was administered intratumorally at a dose of 10 nmol per mouse every other day, while the control group received an equal amount of Control‐ASO injection. The administration continued until the day13 post‐tumor formation. After establishing the PANC‐1 and BxPC3 cell liver metastasis model, the anti‐tumor metastasis efficacy of STAU2‐ASO was evaluated. STAU2‐ASO was administered via tail vein injection at a dose of 10 nmol per mouse every other day, while the control group received an equal amount of Control‐ASO injection. The administration continued until the day13 post‐tumor formation. in vivo imaging was performed on the mice on day 7 and day14, and the mice were euthanized after the procedures on day14. Their livers were harvested, and tissue fluorescence images were taken.

### Immunohistochemistry

Tumors and critical organs from PDAC tumor‐bearing mice were fixed in 4% PFA followed by embedded in paraffin wax, sectioned in slides. Sections were incubated with 3% H_2_O_2_ for 10 min to quench endogenous peroxidase activity followed by antigen retrieval using the unmasking solution. Nonspecific binding was blocked with 2% goat serum for 30 min. These sections were then subjected to Ki67 and hematoxylin and eosin (H&E) staining. Organs from mice in acute toxicity study were carried out for H&E staining.

### Statistical Analysis

For the comparison of differences between the two groups, an unpaired two‐tailed student's *t*‐test was conducted using GraphPad Prism 8 software. As for bioinformatics analysis, both the student's *t*‐test and the Wilcoxon test were employed to compare continuous variables between the two groups. Survival analysis for patients was performed using Kaplan–Meier plots and log‐rank tests. The mean values ± standard deviation (SD) were presented, along with the number of replicates indicated in the figure legends. A significance level of *p* < 0.05 was considered statistically significant.

## Conflict of Interest

The authors declare no conflict of interest.

## Author's Contributions

J.D., H.S. and J.J. equally contributed to this work. P. Y. and X. W. conceived the study. X. W., J. D., H. S., and W. K. designed the research. J. D. and H. S. performed most of the experiments. J. J., J. L., Z. S., X. W., B. L., Y. H. and W. M. conducted the bioinformatics analysis. C. S., K. Y., Y. Z. and L. W. performed data collection and analysis. All authors analyzed the data and discussed the article. J. D., H. S. and X. W. wrote the manuscript, while P. Y., X. W., W.K. and S‐Q. L. reviewed and revised it. P. Y., X. W. and W. K. supervised the study.

## Supporting information



Supporting Information

Supporting Information

Supporting Information

Supporting Information

## Data Availability

Research data are not shared.
