# Supplementary material for: ASO Therapy Targeting STAU2 to Inhibit Pancreatic Ductal Adenocarcinoma Progression and Metastasis by Regulating the PALLD‐Mediated EMT Signaling Pathway
Source: Adv Sci (Weinh). 2025 Jun 20;12(33):e06718. doi: 10.1002/advs.202506718 (PMC12412546; doi:10.1002/advs.202506718)
Supplement: Supplementary file 1 — Supporting Information [file ADVS-12-e06718-s004.docx]

Supplementary Information

ASO Therapy Targeting STAU2 to Inhibit Pancreatic Ductal Adenocarcinoma Progression and Metastasis by Regulating the PALLD-Mediated EMT Signaling Pathway

Jiayu Ding^a, b, #^, Hao Shen^a, b, #^, Jiaying Ji^a, b, #^ Jiaxing Li^a, b^, Zhongrui Shi^a, b^, Xuejiao Wang^a, b^, Bangbang Li^a, b^, Yi Hou^a, b^, Wenjian Min^a, b^, Chengliang Sun^a, b^, Kai Yuan^a, b^, Yasheng Zhu^a, b^, Liping Wang^a, b^, Shun-Qing Liang ^c. *^, Wenbin Kuang^a, b, *^, Xiao Wang^a, b, d, *^ and Peng Yang^a, b, d, *^

*^a^State Key Laboratory of Natural Medicines, China Pharmaceutical University, Nanjing 210009, China*

*^b^Department of Medicinal Chemistry, School of Pharmacy, China Pharmaceutical University, Nanjing 211198, China*

*^c^Department of Medicine, University of Minnesota Twin Cities, Minneapolis, MN 55455, USA*

*^d^Institute of Innovative Drug Discovery and Development, China Pharmaceutical University, Nanjing 211198, China*

^#^These authors made equal contributions to this work.

*Corresponding authors: P.Y. Email: pengyang@cpu.edu.cn; #639 Longmian Avenue, Jiangning District, Nanjing, 211198, P. R. China. X.W. xiaowang@cpu.edu.cn, W.K. 1520220107@cpu.edu.cn, or S.L. lian0198@umn.edu.


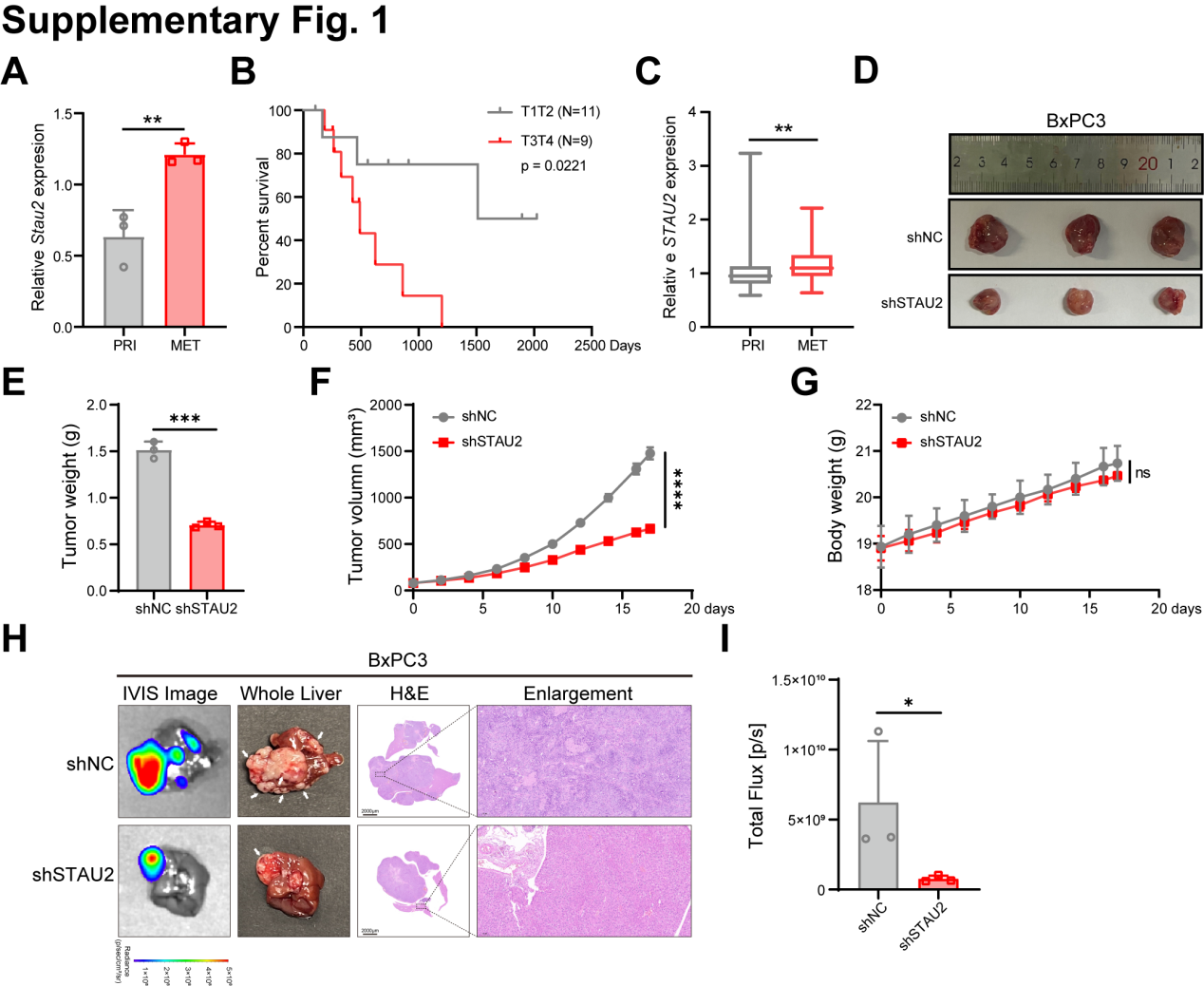


**Figure S1. STAU2 is highly expressed in metastatic PDAC.** (A) Analysis of data from GSE162791 mouse pancreatic ductal adenocarcinoma showed that STAU2 was highly expressed in metastatic pancreatic ductal adenocarcinoma compared to primary pancreatic ductal adenocarcinoma. Data represent the mean ± SD, n = 3. Statistical analysis was performed using two-tailed unpaired student’ s t-test. (B) Clinical samples were divided into early pancreatic ductal adenocarcinoma T_1_T_2_ group and advanced pancreatic adenocarcinoma T_3_T_4_ group according to T stage. KM curve showed that the survival rate of advanced pancreatic ductal adenocarcinoma was significantly lower than that of early pancreatic ductal adenocarcinoma. Statistical analysis was performed using log-rank test. (C) The relative STAU2 expression in primary PDAC and metastatic PDAC from GSE154778 single-cell RNA sequencing data. Data represent the mean ± SD. Statistical analysis was performed using two-tailed unpaired student’ s t-test. (D - F) BALB/c nude mice subcutaneously transplanted with shSTAU2 cells and shNC BxPC-3 cells. Tumor volumes were measured every 2 days (F) , and after mice were euthanized, tumors were excised, photographed (D) and weight (E). (G) No significant difference in the body weight of mice between shNC group and shSTAU2 group during the experimental period. Data represent the mean ± SD, n = 3 mice in each group. Statistical analysis was performed using two-tailed unpaired student’s t-test. (H) Representative bioluminescent images (H, column of IVIS image), photographs (H, column of whole liver, arrows point to the tumor nodules) and HE staining with enlargement (H, columns of HE and enlargement) are shown, respectively. Scale bar, 2000 μm (H&E); 100 μm (enlargement). (I) Quantified after tumor formation in model of PDAC with liver metastasis. Data represent the mean ± SD, n = 3 in each group. Statistical analysis was performed using two-tailed unpaired student’s t-test.*, p < 0.05; **, p < 0.01; ***, p < 0.001; ****, p < 0.0001.


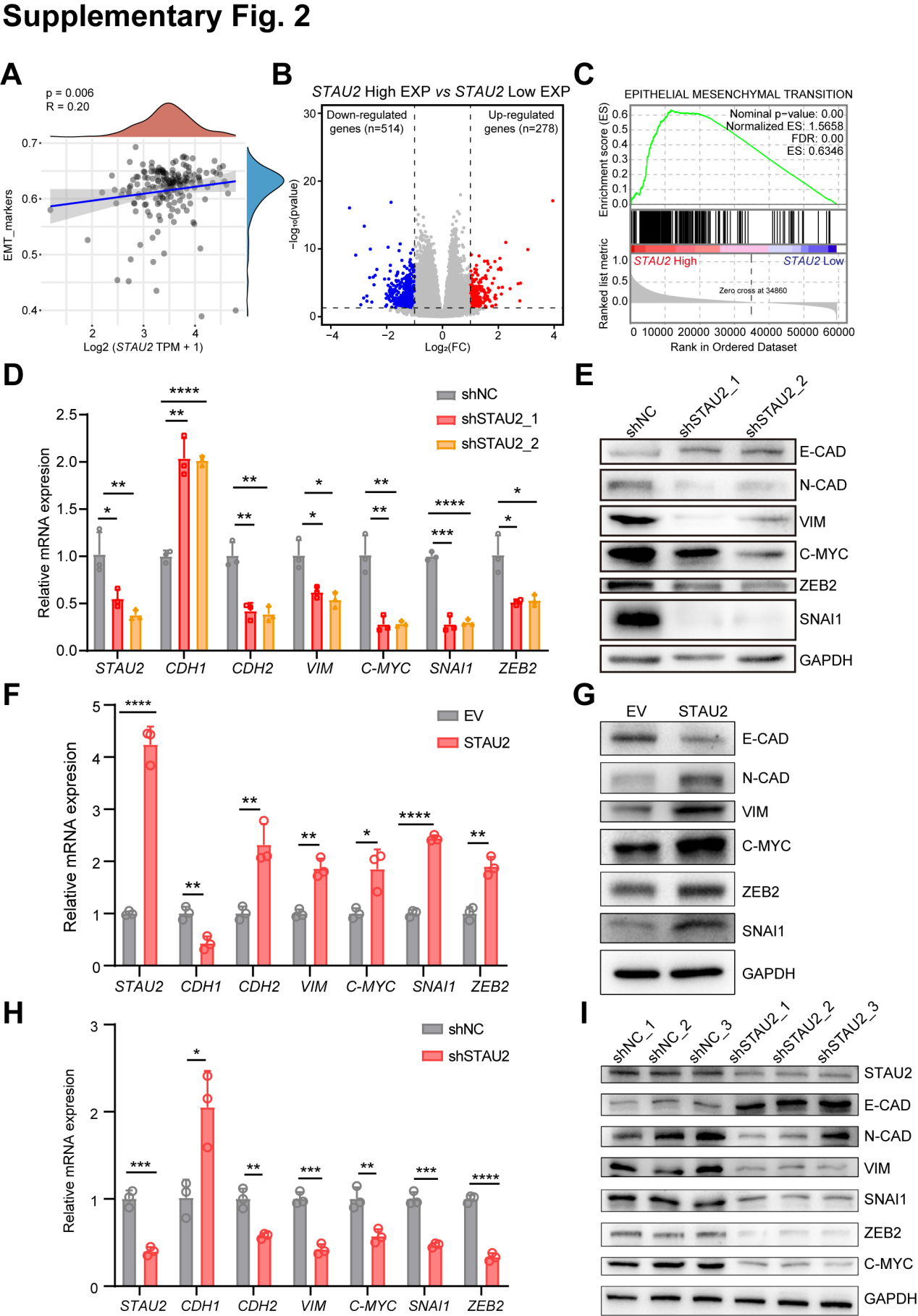


**Figure S2.** **STAU2 is involved in the regulation of epithelial-mesenchymal transition pathway.** (A) STAU2 expression was positively correlated with EMT pathway markers in pancreatic adenocarcinoma by the ssGSEA method. (B) Volcano figure showing the significant different expression genes between high STAU2 expression PDAC patients and low STAU2 expression PDAC patients. (C) GESA analysis revealed the “HALLMARK_EPITHELIAL_MESENCHYMAL_TRANSITION” is significant enriched in high STAU2 expression PDAC patients. (D - E) RT-qPCR (D) and western blot (E) analysis of STAU2 and EMT pathway marker genes expression in BxPC3 cells transfected with shNC and two independent shSTAU2 constructs. Data represent the mean ± SD, n = 3. Statistical analysis was performed using two-tailed unpaired student’ s t-test. (F - G) RT-qPCR (F) and Western blot (G) analysis of STAU2 and EMT pathway marker genes expression in BxPC3 cells that were overexpressed with EV and STAU2. Data represent the mean ± SD, n = 3. Statistical analysis was performed using two-tailed unpaired student’ s t-test. (H - I) RT-qPCR (H) and western blot (I) analysis of STAU2 and EMT pathway marker genes expression in tumor tissues of shNC and shSTAU2 groups from CDX models constructed by BxPC3. Data represent the mean ± SD, n = 3 in each group. Statistical analysis was performed using two-tailed unpaired student’s t-test.*, p < 0.05; **, p < 0.01; ***, p < 0.001; ****, p < 0.0001.

**
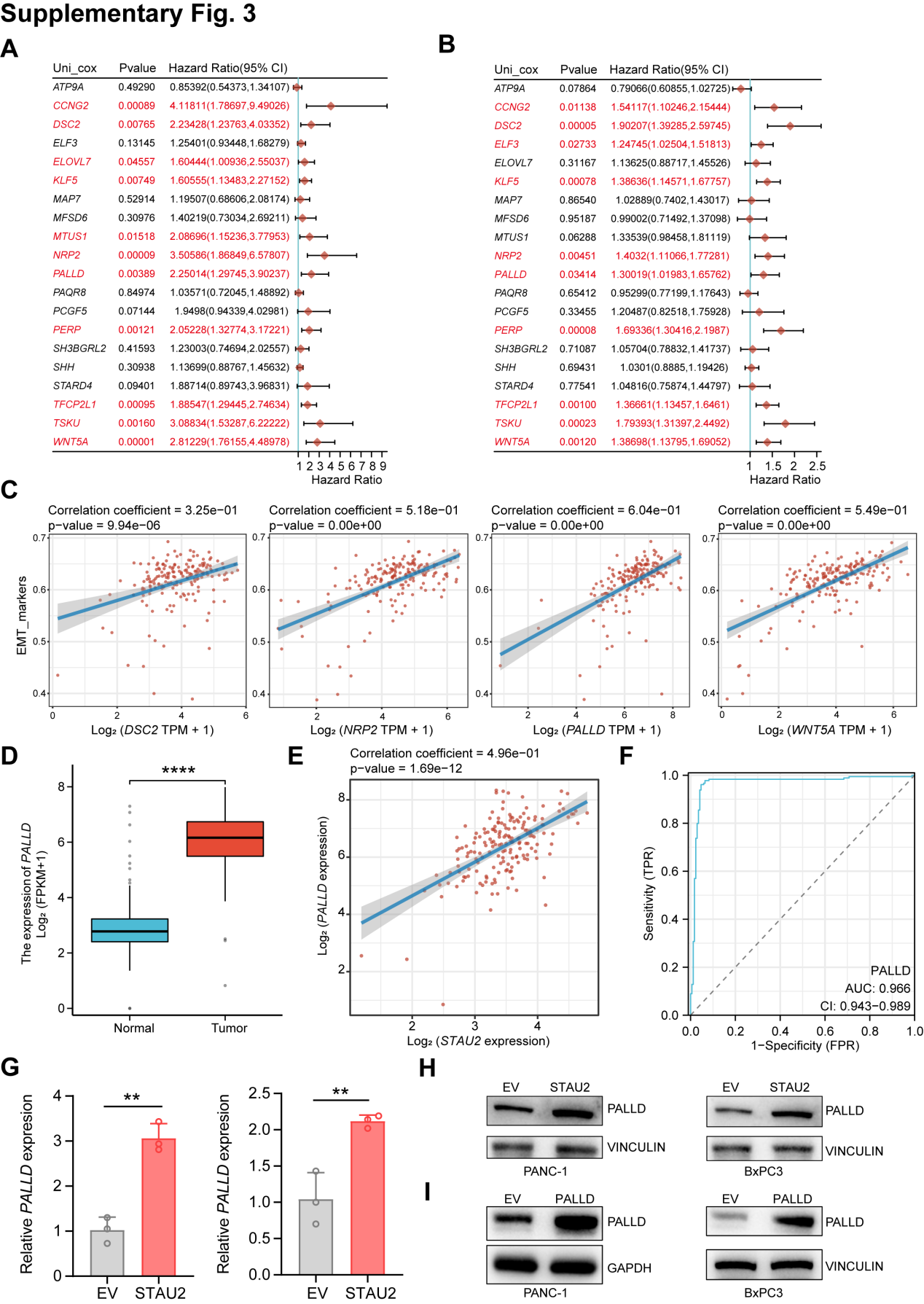
**

**Figure S3. Screening and identification of STAU2 binding RNA targets****.**

(A) Overall survival analysis of STAU2 binding target RNAs. (B) Disease free survival analysis of STAU2 binding target RNAs. (C) Relationship between STAU2 target gene expression associated with cancer metastasis and EMT pathway markers in pancreatic ductal adenocarcinoma by ssGSEA method. (D) Comparison of PALLD expression between PDAC tumor samples (n = 179, from TCGA) and normal samples (n = 171, from TCGA & GTEx). Statistical analysis was performed using wilcoxon rank sum test, *p* < 0.0001. (E) The expression of STAU2 was positively correlated with PALLD in PDAC. (F) Receiver operating characteristic (ROC) curve evaluating the diagnostic potential of PALLD expression in PDAC, AUC = 0.966. (G) RT-qPCR analysis of PALLD mRNA levels in PANC-1 and BxPC3 cells overexpressing EV or STAU2. (H) Western blot analysis of PALLD protein expression in PANC-1 and BxPC3 cells overexpressing EV or STAU2. (I) Western blot assessment of PALLD protein levels in PANC-1 and BxPC3 cells ectopically expressing EV or PALLD. Data represent the mean ± SD, n = 3 in each group. Statistical analysis was performed using two-tailed unpaired student’s t-test.*, p < 0.05; **, p < 0.01; ***, p < 0.001; ****, p < 0.0001.

**
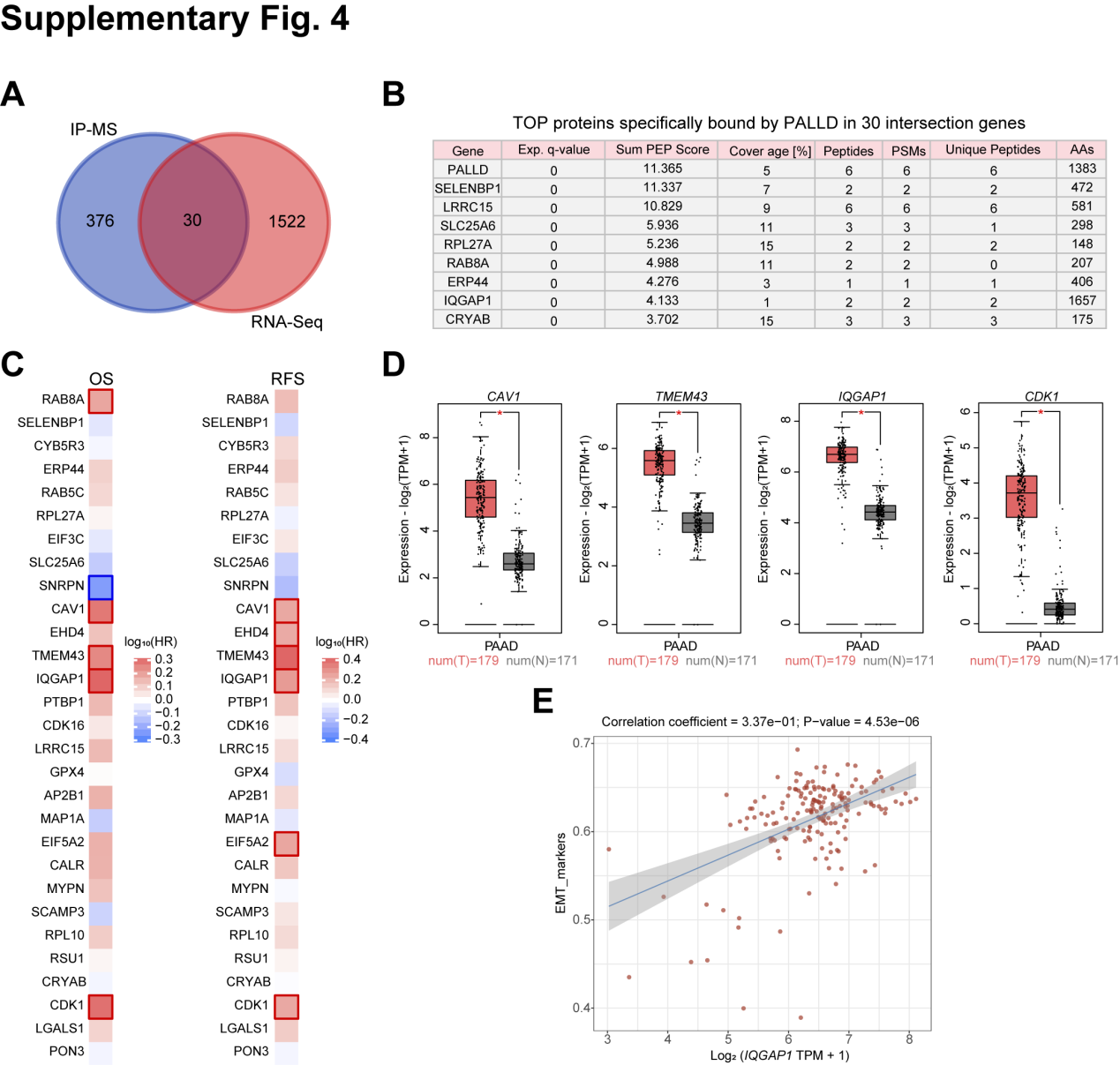
**

**Figure S4. PALLD interacts with IQGAP1 to regulate the EMT pathway.**

(A) Proteins specifically binding to PALLD identified by IP-MS, which were intersected with 1,552 significantly down-regulated genes identified by RNA-Seq, resulted in 30 key genes that are potential downstream targets of PALLD. (B) Top proteins specifically bound by PALLD in 30 key genes. (C) Overall and recurrence-free survival analysis of 30 key genes. (D) Comparison of genes expression between PDAC tumor samples (n = 179, from TCGA) and normal samples (n = 171, from TCGA & GTEx). (E) Relationship between IQGAP1 expression associated with cancer metastasis and EMT pathway markers in pancreatic ductal adenocarcinoma by ssGSEA method. Statistical analysis was performed using two-tailed unpaired student’s t-test. *, p < 0.05; **, p < 0.01; ***, p < 0.001; ****, p < 0.0001.

**
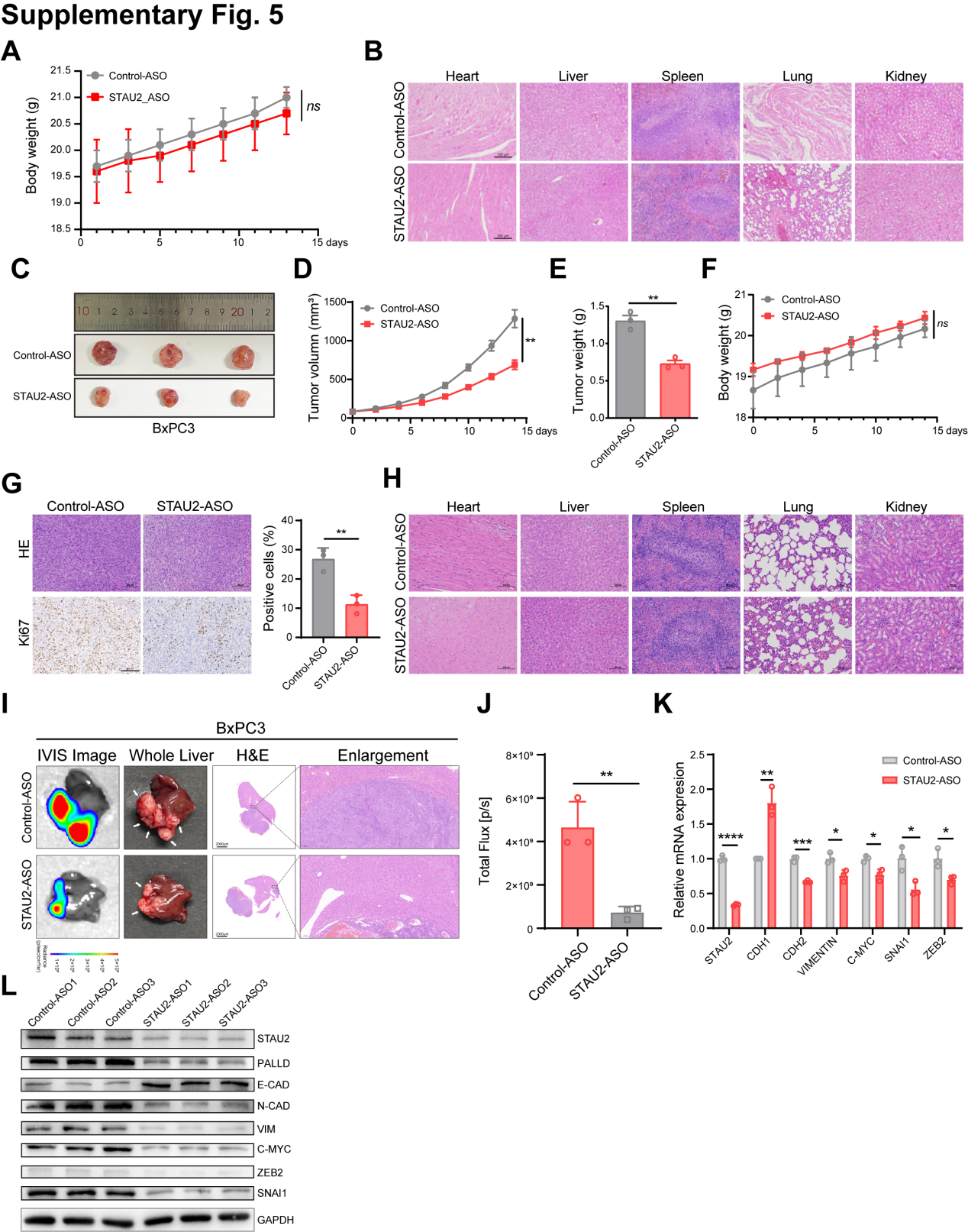
**

**Figure S5. STAU2-ASO significantly inhibited the development and metastasis of PDAC with a certain safety profile.**

(A) No significant difference in the body weight of PANC-1 xenograft model between groups treated with Control-ASO or STAU2-ASO during the experimental period. Data represent the mean ± SD, n = 3 mice in each group. (B) HE immunohistochemical staining was performed on the heart, liver, spleen, lung and kidney of PANC-1 xenograft model treated with Control-ASO and STAU2-ASO. Scale bar, 100 μm. (C - E) BALB/c nude mice subcutaneously transplanted with BxPC3 cells were treated with Control-ASO or STAU2-ASO. Tumor volumes were measured every 2 days (D) , and after mice were euthanized, tumors were excised, photographed (C) and weight (E). (F) No significant difference in the body weight of BxPC3 xenograft model treated with Control-ASO or STAU2-ASO during the experimental period. Data represent the mean ± SD, n = 3 mice in each group. Statistical analysis was performed using two-tailed unpaired student’s t-test. (G) Quantitative analysis of the ratio of Ki67 positive cells in three independent tumors from the STAU2-ASO treatment group and the Control-ASO treatment group. Data represent the mean ± SD, n = 3 mice in each group. Statistical analysis was performed using two-tailed unpaired student’s t-test. Scale bar, 100 μm. (H) HE immunohistochemical staining was performed on the heart, liver, spleen, lung and kidney of BxPC3 xenograft model treated with Control-ASO and STAU2-ASO. Scale bar, 100 μm. (I) Representative bioluminescent images (I, column of IVIS image), photographs (I, column of whole liver, arrows point to the tumor nodules) and HE staining with enlargement (I, columns of HE and enlargement) are shown, respectively. Scale bar, 2000 μm (H&E); 100 μm (enlargement). (J) Quantified after tumor formation in model of PDAC with liver metastasis. (K - L) RT-qPCR (K) and western blot (L) analysis of EMT pathway markers in a liver metastasis PDAC model treated with Control-ASO or STAU2-ASO. Data represent the mean ± SD, n = 3 in each group. Statistical analysis was performed using two-tailed unpaired student’s t-test.*, p < 0.05; **, p < 0.01; ***, p < 0.001; ****, p < 0.0001.
